# Tracking 21^st^ century anthropogenic and natural carbon fluxes through model-data integration

**DOI:** 10.1038/s41467-022-32456-0

**Published:** 2022-09-26

**Authors:** Selma Bultan, Julia E. M. S. Nabel, Kerstin Hartung, Raphael Ganzenmüller, Liang Xu, Sassan Saatchi, Julia Pongratz

**Affiliations:** 1 Department of Geography, Ludwig-Maximilians-Universität, Munich, Germany; 2 Max Planck Institute for Meteorology, Hamburg, Germany; 3 Max Planck Institute for Biogeochemistry, Jena, Germany; 4 Jet Propulsion Laboratory, California Institute of Technology, Pasadena, CA USA; 5 Present Address: Deutsches Zentrum für Luft- und Raumfahrt, Institut für Physik der Atmosphäre, Oberpfaffenhofen, Germany; 6 Present Address: Pachama Inc., San Francisco, CA USA

**Keywords:** Carbon cycle, Biogeography

## Abstract

Monitoring the implementation of emission commitments under the Paris agreement relies on accurate estimates of terrestrial carbon fluxes. Here, we assimilate a 21^st^ century observation-based time series of woody vegetation carbon densities into a bookkeeping model (BKM). This approach allows us to disentangle the observation-based carbon fluxes by terrestrial woody vegetation into anthropogenic and environmental contributions. Estimated emissions (from land-use and land cover changes) between 2000 and 2019 amount to 1.4 PgC yr^−1^, reducing the difference to other carbon cycle model estimates by up to 88% compared to previous estimates with the BKM (without the data assimilation). Our estimates suggest that the global woody vegetation carbon sink due to environmental processes (1.5 PgC yr^−1^) is weaker and more susceptible to interannual variations and extreme events than estimated by state-of-the-art process-based carbon cycle models. These findings highlight the need to advance model-data integration to improve estimates of the terrestrial carbon cycle under the Global Stocktake.

## Abstract (web summary)

Accurate estimates of carbon fluxes are important to our understanding of the carbon cycle. Here, via model-data integration, the authors disentangle anthropogenic and environmental carbon flux contributions of terrestrial woody vegetation, and find that environmental processes are weaker and more susceptible to interannual variations and extreme events in the 21st century than previously estimated.

## Introduction

Environmental change is altering the global balance between CO_2_ emissions and uptakes by terrestrial ecosystems. The natural carbon sinks in terrestrial vegetation and soils provide an immense buffer for anthropogenic emissions, currently sequestering about one-third of fossil and land-use change CO_2_ emissions^1^. Opposing effects on the strength of the terrestrial carbon sinks, such as increased plant productivity through CO_2_ fertilization^2^ and enhanced wildfires triggered by pronounced droughts^3^, lead to large uncertainties when estimating present and future dynamics of the natural carbon sinks^4^. Reducing those uncertainties through analyzing the individual contributions of anthropogenic and environmental processes to the global carbon cycle is one of the main aims of the annually updated Global Carbon Budget (GCB), published by the Global Carbon Project^1^. Within the scope of the GCB, the net land-atmosphere exchange of CO_2_ is defined as the sum of (1) anthropogenic fluxes from land-use and (land-use induced) land cover change activities (LULCC), including management, and (2) fluxes due to environmental processes (e.g., effects of increased atmospheric CO_2_ levels and N-deposition, pests, wildfires, altered precipitation patterns). The first term is called *E*_*LUC*_ and is estimated with semi-empirical bookkeeping models (BKMs), whereas the second term is referred to as the natural terrestrial carbon sink, *S*_LAND_, which is estimated with process-based dynamic global vegetation models (DGVMs).

For both *E*_LUC_ and *S*_LAND_, there is a large spread among model estimates. *E*_LUC_ is estimated to be 1.1 ± 0.7 PgC yr^−1^ for 2011–2020, i.e., has an uncertainty of ±64% (for one standard deviation). The DGVM estimate for *S*_LAND_ for the same time frame has an uncertainty of ±19%^1^. BKMs commonly simulate emissions due to LULCC in the absence of environmental influences by combining assumptions on the amount of carbon contained in vegetation and soils with empirical decay functions, describing their response to LULCC. DGVMs additionally account for environmental effects on the different carbon pools and simulate biogeochemical processes such as photosynthesis^5^.

The main sources of uncertainty depend on the model type. For BKMs, different assumptions regarding the amount of contained carbon per unit area (=carbon density) contribute substantially to the uncertainty in *E*_LUC_^6^. Moreover, carbon densities of soils and vegetation in BKMs are typically based on contemporary carbon stocks, which inevitably introduces an error when simulating past (i.e., the period prior to the data record of the underlying carbon densities) *E*_LUC_ (“bookkeeping error”)^7^. For DGVMs, different parameterizations and whether and how vegetation and soil processes are captured lead to a large spread in the estimated soil and vegetation carbon stocks (vegetation carbon ±55% of the average for eight DGVMs from the TRENDY Model-Intercomparison Project (https://blogs.exeter.ac.uk/trendy/, last access: 11 April 2022); see Table 1)^8^.

**Table 1:** Comparison of global living biomass (above- plus belowground) carbon stocks and associated fluxes (positive for uptake and negative for release) from this study compared to a range of other recent studies

			Living biomass carbon stocks and fluxes (AGB + BGB)			
	Dataset	Period	Stocks (PgC)	Flux type	Net flux (PgC yr^-1^)	IAV of net flux
This study*	transient woody biomass carbon^a,e^	2000–2019	399 ± 2	*E*_LUC,*B*_ + *S*_LAND,*B*_	−0.6 ± 0.0	2.4 ± 0.0
	fixed woody biomass carbon^a^	2000–2019	382 ± 2	*E*_LUC,*B*_	−2.0 ± 0.0	0.3 ± 0.0
Hansis et al.^5^^a^		2000–2019	506	*E*_LUC,*B*_	−1.2	0.3
Xu et al.^16^•^a,e^		2000–2019	381 ± 2	*E*_LUC,*B*_ + *S*_LAND,*B*_	0.3	6.1
TRENDY v8 #	S3^a,e^ (transient environmental conditions)	2000–2018	368 ± 204	*E*_LUC,*B*_ + *S*_LAND,*B*_	0.4 ± 0.6	2.2 ± 2.9
	S5^a^ (fixed environmental conditions)	2000–2018	434 ± 237	*E*_LUC,*B*_	−0.8 ± 0.6	0.7 ± 0.2
Liu et al.^53^ $^a,e^		1998–2002	362	-		
Tagesson et al.^22^ $^a,e^		1993–2012	353	*E*_LUC,*B*_ + *S*_LAND,*B*_	-	
Erb et al.^23^ (Compilation of datasets)^a,e^		multiple	407-476	-		
Spawn et al.^24^^a,e^		2010	409	-		

Interannual variability (IAV) is calculated as the ratio of the standard deviation (SD) to the mean. Note that the IAV estimates presented in ref. 16 are calculated as the standard deviation and therefore differ from our estimates. Error estimates are given as the mean of eight TRENDY DGVMs ± 1 SD (#) resp. as the mean from two threshold approaches (see Methods) ±an error of 0.5% propagated from ref. 16 to our woody vegetation carbon estimates (*). Note that for estimates that only consider aboveground biomass, roughly 20–60% needed to be added to account for belowground biomass carbon^16^. Note that, to avoid errors from the rounding of numbers in the table, percentage values in the main text were calculated from unrounded numbers.

$ Estimate only includes aboveground biomass carbon.

• Estimate only includes woody biomass carbon.

^e^ Estimate includes environmental influences.

^a^ Estimate includes anthropogenic influences.

Observational estimates of global vegetation carbon stock from existing datasets^9,10^ and upcoming satellite missions (e.g., ESA BIOMASS mission^11^) offer large potentials for reducing the mentioned model uncertainties by constraining models with observations^12,13^. However, there are limitations to all satellite-based estimates of global vegetation carbon fluxes for terrestrial carbon budget analyses. Some of the major limitations are: the restriction to gross fluxes/sub-component fluxes of *E*_LUC_ (e.g., only carbon emissions from deforestation or carbon uptakes after the abandonment of agricultural lands) are captured; the difficulty to distinguish anthropogenic from environmental fluxes and the restriction to committed fluxes^14^. In committed fluxes, biomass loss is assumed equal to emissions to the atmosphere, unless additional assumptions are applied that track the fate of carbon on site and in products over time, as in legacy fluxes^15^.

Here, we propose an approach that overcomes the mentioned limitations of BKMs and satellite-based estimates by allowing us to decompose observationally constrained estimates of carbon stocks into anthropogenic (*E*_LUC_) and environmental (*S*_LAND_) contributions. We only count fluxes resulting from direct anthropogenic activities in the form of LULCC towards anthropogenic processes. These include carbon uptakes due to regrowth after wood harvesting and abandonment of agricultural lands and carbon emissions due to forest clearing, wood harvesting, etc. Indirect anthropogenic influences (e.g., effects of increasing CO_2_ on plant productivity) are defined as environmental processes. Our analysis is based on the recently published time series of global woody vegetation carbon densities for 2000–2019 by ref. 16. The observation-based time series is assimilated into the BKM BLUE (“Bookkeeping of Land Use Emissions”)^5^, which is one of three BKMs used in the GCB. We apply our approach to analyse the implications of considering environmental processes on the estimated *E*_LUC_. Furthermore, we assess uncertainties of the land cover and plant functional type distribution in BLUE. Lastly, we provide observation-based *S*_LAND_ estimates for woody vegetation, which are subsequently compared to DGVMs from the TRENDY project (v8)^8^.

## Results

### A model-data integration framework for separating anthropogenic and environmental carbon fluxes

Our methodological framework, as shown in Supplementary Fig. 1, is based on the BKM BLUE and the time series of woody vegetation carbon densities from ref. 16. In its default setup, BLUE simulates LULCC emissions in the absence of environmental influences. The carbon densities of vegetation and soils (see Methods) on different land cover types (primary land, secondary land, cropland and pasture) and plant functional types (PFTs) are based on fixed contemporary values^17^. Over time, the amount of carbon stored in the terrestrial biosphere is altered by LULCC prescribed from external data (here: the Land-Use Harmonization 2 (LUH2)^18^ dataset). Recovery and decay of soil and vegetation carbon follow fixed contemporary rates (see ref. 17 and Methods).

The dataset by ref. 16 provides annual estimates of carbon densities in living woody vegetation (i.e., trees and shrubs) for the period 2000–2019. The dataset was generated by integrating data from spaceborne LiDAR, RADAR, optical imagery, airborne laser scanning and ground inventory data in a spatio-temporal machine learning algorithm. The algorithm was trained by a large number of samples derived from LiDAR measurements of vegetation structure converted to aboveground and belowground woody vegetation carbon densities using allometric models^16^. We assimilate this dataset in BLUE in several steps to calculate *S*_LAND_: We (1) distribute the grid cell-based (i.e., average per grid cell) biomass carbon densities of ref. 16 between sub-pixel fractions for the different land cover types and PFTs in BLUE, (2) define upper thresholds for the exclusion of unrealistic biomass carbon densities that arise from inconsistencies between the dataset by ref. 16 and the model assumptions, and (3) interpolate “missing values” (i.e., values that were excluded according to the chosen threshold approach) (see Methods). We employ two different simulation setups to isolate environmental carbon fluxes (*S*_LAND_) by terrestrial woody vegetation (4). The first setup (4a) relies on transient biomass carbon densities, i.e., the biomass carbon densities from ref. 16 are assimilated into BLUE at each time step (i.e., each year). In this setup, carbon stocks of woody biomass between two time steps are affected by anthropogenic and environmental drivers. The second setup (4b) is based on fixed biomass carbon densities from the year 2000. In this setup, the biomass carbon densities from ref. 16 are assimilated into BLUE for the year 2000 and the carbon stocks are in the subsequent time steps only altered by LULCC, i.e., only anthropogenic processes are considered. We 5) define the difference in the annual change of woody biomass carbon between the transient and the fixed simulation setup as the woody terrestrial biomass carbon sink (=*S*_LAND,*B*_). Our definition is consistent with the GCB in the sense that it defines *S*_LAND,*B*_ as the sum of all carbon sources and sinks due to environmental processes on any type of land (i.e., managed and unmanaged). However, there are substantial differences between our estimates and the GCB estimates, including differences in the assumed land cover distribution (see following explanation on the “loss of additional sink capacity”) and the restriction of our estimates to woody vegetation and to living biomass, excluding litter, dead wood and soil dynamics. Contrary to the GCB, our estimated carbon fluxes from woody vegetation due to LULCC (*E*_LUC,*B*_) and *S*_LAND,*B*_ should not be used to create a balance to derive the net exchange of carbon between the land and the atmosphere, since *E*_LUC,*B*_ only captures fluxes to/from the atmosphere from/to woody vegetation, whereas *S*_LAND,*B*_ also includes fluxes that are in reality delayed (to the atmosphere) due to the deposition of carbon to litter and soil carbon pools. In our approach, carbon releases from woody vegetation are positive, whereas uptakes of carbon by woody vegetation are negative.

Our approach introduces various important novelties compared to DGVMs and to BKMs with fixed contemporary carbon densities. Compared to DGVMs, our approach has the advantage that estimates for *S*_LAND,*B*_ and *E*_LUC_ are estimated on transient, present-day land cover distribution, and do therefore not include the “loss of additional sink capacity” (LASC). The LASC implies that *S*_LAND_ from DGVM simulations without LULCC under transient environmental conditions and under pre-industrial land cover distribution is larger than under present-day land cover distribution. This is due to larger forested areas under pre-industrial land cover compared to present-day land cover, which allows for more carbon accumulation caused by favorable environmental conditions (e.g., increasing atmospheric CO_2_)^19^. Similarly, a recent study by ref. 19 suggests that calculating *E*_LUC_ as the difference between DGVM simulations with and without LULCC (i.e., under pre-industrial land cover) under transient environmental conditions leads to a 40% larger *E*_LUC_ for 2009–2018 compared to the pre-industrial control simulation, which is attributable to the LASC. Furthermore, the bookkeeping error is resolved for *E*_LUC,*B*_ after 2000 due to the assimilation of observed woody biomass carbon densities. Lastly, our approach considers all impacts on carbon fluxes related to woody vegetation, including processes that are commonly not considered in model-based approaches (e.g., forest degradation caused by environmental processes).

Throughout our analysis, we use slightly different time frames for aggregating the data from our BLUE simulations. The data on *E*_LUC_ and on biomass carbon stocks is aggregated for the entire time series, i.e., 2000–2019. Fluxes that are calculated from annual changes in biomass carbon, including *S*_LAND,*B*_, are available for 2001–2019. However, for any direct comparisons with only the TRENDY DGVMs, we restrict our *S*_LAND,*B*_ estimates to the time frame of the TRENDY data on *S*_LAND,*B*_ (2001–2018).

### Anthropogenic effects (LULCC) on global woody vegetation carbon

To assess the general behavior of the BKM using updated woody biomass carbon stocks from observations, we compare the results of our fixed woody biomass carbon simulations to the default setup (of BLUE) and other models, which follow the classical bookkeeping approach (i.e., exclude environmental influences). The default setup of BLUE is based on carbon densities from ref. 17 and is referred to as ref. 5 in Table 1–3. On a global scale, *E*_LUC_ between 2000 and 2019 for the fixed woody biomass runs is on average 0.2 PgC yr^−1^ (13%) lower than the estimate with the default setup. This brings our updated *E*_LUC_ estimate closer to the other BKMs used in the GCB^20,21^ and to the multi-model average of the TRENDY simulations with fixed present-day carbon densities^19^ (Table 2). The spread between the BKMs is reduced by 43% (BLUE minus OSCAR^20^) resp. 29% (BLUE minus H&N^21^), whereas the difference to the TRENDY multi-model average (1.4 PgC yr^−1^) is reduced by 88% compared to the default BLUE setup.

**Table 2:** Comparison of estimated global carbon flux from land-use and (land-use induced) land cover change (positive into atmosphere) from this study compared to a range of other recent studies. Interannual variability (IAV) is calculated as the ratio of the standard deviation (SD) to the mean

			Carbon flux from land-use and land cover change (*E*_LUC_)		
	Dataset	Period	Cumulative (PgC)	Net flux (PgC yr^−1^)	IAV of net flux
This study *	transient woody biomass carbon^a,e^	2000–2019	57	2.8	0.2
	fixed woody biomass carbon^a^	2000–2019	27	1.4	0.2
Hansis et al.^5^^a^		2000–2019	32	1.6	0.2
Xu et al.^16^^a,e^		2001–2019	88	4.6	0.1
Houghton et al.^21^^a^		2000–2019	17	0.9	0.2
Gasser et al.^20^	transient environmental conditions^a,e^	2000–2018	25	1.3	0.1
	fixed environmental conditions^a^	2000–2018	21	1.1	0.1
TRENDY v8#	S2-S3^a,e^ (transient environmental conditions)	2000–2018	29 ± 9	1.5 ± 0.4	0.3 ± 0.1
	S6-S5^a^ (fixed environmental conditions)	2000–2018	26 ± 9	1.3 ± 0.5	0.4 ± 0.1

Note that the IAV estimates presented in ref. 16 are calculated as the standard deviation and therefore differ from our estimates. Error estimates are given as the mean of eight TRENDY DGVMs ± 1 SD (#) resp. as the mean from two threshold approaches (*) (see Methods). Note that, to avoid errors from the rounding of numbers in the table, percentage values in the main text were calculated from unrounded numbers.

^e^ Estimate includes environmental influences.

^a^ Estimate includes anthropogenic influences.

*E*_LUC_ estimated from the transient woody biomass carbon simulations amounts to 2.8 PgC yr^−1 ^ for 2000–2019. The large difference in the fixed woody biomass carbon estimate for *E*_LUC_ is mainly related to higher biomass carbon stocks in the transient simulations (probably strongly driven by the effect of enhanced plant productivity under increasing CO_2_)^2,22^. In the fixed carbon density simulation, vegetation on cleared or harvested areas is recovering/slowly regrowing in the years after the respective land-use event. In the transient carbon density simulation, the effect of higher CO_2_ on plant productivity, together with other environmental influences, leads to increased carbon uptake by woody vegetation. On managed lands, increasing atmospheric CO_2_ can lead to a quicker recovery of vegetation after clearing/harvesting. Consequently, the higher biomass carbon in the transient simulation can lead to increased emissions upon wood harvesting and clearing but also to increased carbon uptake due to faster regrowth of vegetation on managed lands. To identify the drivers behind the higher net *E*_*LUC*_ in the transient woody biomass simulations, we analysed *E*_*LUC*_ for the major land-use transitions in BLUE (clearing, harvest, and abandonment) (Supplementary Table 1). Accordingly, emissions from clearing and wood harvesting are on average 1.7 PgC yr^−1^ higher in the transient woody carbon density setup than in the fixed woody carbon density setup, which is not compensated by the increased carbon uptakes (i.e., negative flux from the atmosphere to the land) due to the quicker vegetation regrowth on abandoned agricultural land under higher CO_2_ concentrations (transient minus fixed: −0.3 PgC yr^−1^). Regional hotspots that make up ~60% of the cumulative increase in *E*_LUC_ in the transient simulation setup are found in Europe, South- and Southeast Asia. Furthermore, Europe and South Asia alone account for the majority (~76%) of the increase in cumulative harvest emissions. Building upon the uncertainty analysis in the Methods, these are all regions where uncertainties due to the LULCC forcing and its implementation in BLUE are high. On different spatial and temporal scales than the ones investigated in our analysis, the effect of larger *E*_LUC_ under transient woody biomass carbon could be reduced or compensated by an increased uptake of carbon due to faster vegetation regrowth under more favorable growing conditions (e.g., due to increasing atmospheric CO_2_ concentrations).

### Environmental effects on global woody vegetation carbon

We analyse similarities and differences between our estimates from BLUE simulations with transient and fixed woody biomass carbon to other model-based and observational estimates. Furthermore, we compare global and regional environmental carbon fluxes in the form of *S*_LAND,*B*_ from our approach to estimates of an ensemble of TRENDY models for the period 2001–2018.

Between 2000 and 2019, we estimate 399 ± 2 PgC contained in global living vegetation (woody and non-woody) in the transient woody biomass carbon simulations vs. 382 ± 2 PgC in the fixed woody biomass carbon simulations. The difference of 17 ± 1 PgC is due to environmental changes. The TRENDY estimates suggest that biomass carbon stocks under fixed climate (S5 setup, see Methods) are 18% higher than under transient climate (S3 setup, see Methods). Similar to our BLUE simulations, this is probably related to the fact that the TRENDY simulations under fixed climate rely on present-day CO_2_ levels, leading to enhanced plant productivity compared to the simulations under a transient climate that also have transient CO_2_ levels^19^. However, the assumption of constant, present-day CO_2_ levels over the whole historical period in the TRENDY S5 simulations leads to a much stronger CO_2_ fertilization effect on vegetation carbon stocks compared to our simulations. The comparison of our estimated vegetation carbon stocks to various other studies (Table 1) shows both BLUE estimates (transient and fixed) are more consistent with the multi-model average of eight TRENDY models (see Methods) and various observation-based datasets^23,24^ than the default setup. Our updated estimates of global forest carbon stocks (Table 3) are also closer to other observation-based estimates^23^ than the estimates from the default setup. The largest differences to the default setup and the biggest improvements concerning the reconciliation with other datasets are found for tropical and boreal forests. In terms of the interannual variability (IAV) of the net carbon fluxes from global woody vegetation (Table 1), we find that the IAV is on average around eight times larger when considering environmental effects on woody biomass carbon. In other words, ~88% (2.1 PgC yr^−1^) of the IAV of the net carbon fluxes from woody biomass (2.4 PgC yr^−1^) carbon is due to environmental effects and their synergies on *E*_LUC_ or conversely ~12% of the IAV (0.3 PgC yr^−1^) is attributable to LULCC (Table 1). The same relation between biomass carbon simulated under fixed vs. transient climate is also shown for the TRENDY simulations, although our estimates suggest a stronger contribution of environmental processes to the IAV of carbon fluxes from vegetation. Between 2001 and 2018, *S*_LAND,*B*_ amounts to −1.6 PgC yr^−1^ (−1.5 PgC yr^−1^ for 2001–2019) based on our BLUE simulations, suggesting a ~13% smaller sink than the TRENDY multi-model average (Supplementary Table 2). There are some important differences between the TRENDY estimates and our BLUE results. First, the TRENDY results do not only include woody vegetation, but also herbaceous plants. Consequently, the IAV of the TRENDY estimates also includes dynamics of non-woody vegetation. However, we expect this effect to be small, since several studies^25,26^ show that the IAV of the terrestrial carbon sink in semi-arid ecosystems is foremost attributable to soil dynamics (as opposed to vegetation dynamics). Second, as mentioned before, our estimates do not include the LASC, as they are based on the present-day land cover distribution. The overestimation of the terrestrial carbon sink strength due to the fixed pre-industrial land cover distribution in the TRENDY simulation is in line with the fact that our BLUE estimate for *S*_LAND,*B*_ shows a smaller sink than the TRENDY multi-model average. Third, the TRENDY models have a much coarser horizontal resolution (0.5^∘^–2.8^∘^) than BLUE (0.25^∘^), which has important implications for the representation of sub-grid scale processes (discussed below).

**Table 3:** Comparison of forest living biomass carbon stocks (above- plus belowground) and associated fluxes (positive for uptake and negative for release) from this study compared to a range of other recent studies

			Living forest biomass carbon stocks and fluxes (AGB + BGB)					
			Stocks (PgC)				Flux type	Net flux (PgC yr^-1^)
	Dataset	Period	Global	Boreal	Temperate	Tropical		Global
This study*	transient woody biomass carbon^a,e^	2000–2019	311 ± 2	81 ± 0	52 ± 0	178 ± 1	*E*_LUC,*B*_ + *S*_LAND,*B*_	−0.4 ± 0.0
	fixed woody biomass carbon^a^	2000–2019	296 ± 1	78 ± 0	48 ± 0	170 ± 1	*E*_LUC,*B*_	−1.7 ± 0.0
Hansis et al.^5^^a^		2000–2019	415	101	52	262	*E*_LUC,*B*_	−1.1
Xu et al.^16^•^a,e^		2000–2019	315	51	55	209	*E*_LUC,*B*_ + *S*_LAND,*B*_	−0.2
Liu et al.^53^ $^a,e^		1998–2002	235	73		162	-	
Tagesson et al.^22^ $^a,e^		1993–2012	235	59	28	148	-	
Erb et al.^23^ (Compilation of datasets)^a,e^		multiple	297–368					-

Error estimates are given as the mean from two threshold approaches (see Methods) ±an error of 0.5% from ref. 16 to our woody vegetation carbon estimates (*). Note that for estimates that only consider aboveground biomass, roughly 20–60% needed to be added to account for belowground biomass carbon^16^.

$ Estimate only includes aboveground biomass carbon.

• Estimate only includes woody biomass carbon.

^e^ Estimate includes environmental influences.

^a^ Estimate includes anthropogenic influences.

**Fig. 1:**
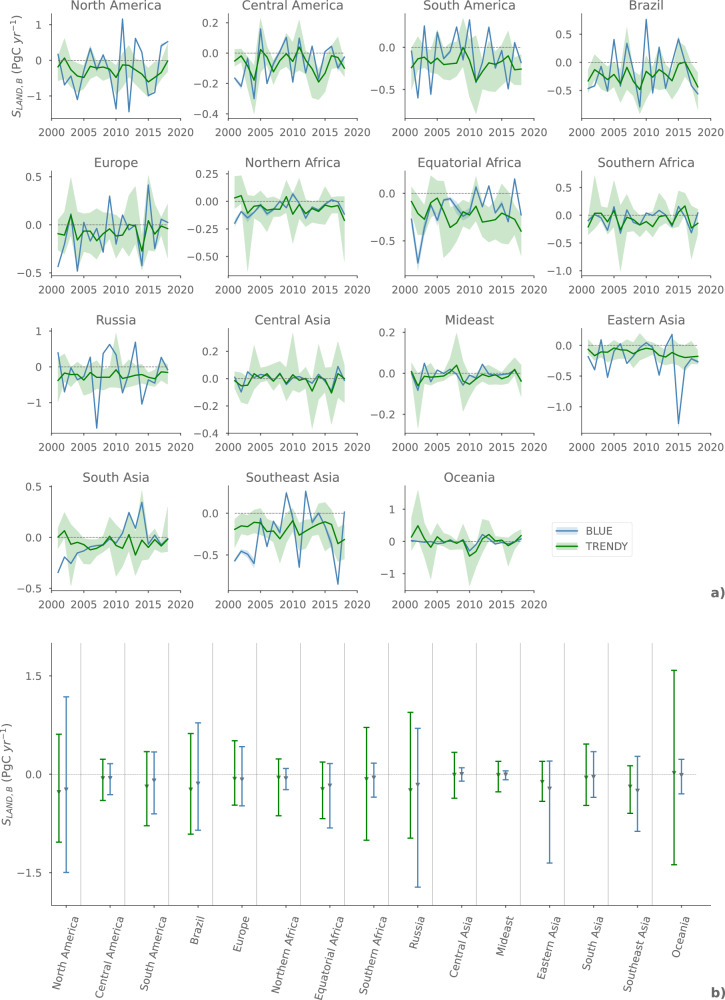
Regional estimates of the natural biomass land sink (*S*_LAND,*B*_) between 2001 and 2018. The figures compare the estimates by 13 DGVMs of the TRENDY model-intercomparison project (v8) vs. our observation-based estimates with BLUE (woody vegetation only). The temporal evolution for each region between 2001 and 2018 is shown in **a**. The lines mark the TRENDY multi-model average resp. the BLUE average from two threshold approaches to exclude unrealistically high woody biomass carbon densities (see Supplementary materials). The TRENDY multi-model range and the range between the BLUE threshold approaches are shown as shaded areas. The variability of *S*_LAND,*B*_, averaged over each region, is presented in **b**: The whiskers extend from the multi-model minimum to the multi-model maximum between 2001 and 2018, and the multi-model average over all years is shown as gray triangles. Uptakes of carbon by vegetation are negative (sinks), whereas releases of carbon by vegetation are positive (sources).

Figure 1 shows *S*_LAND,*B*_ from our BLUE simulations for 15 regions (Supplementary Fig. 2) against the TRENDY estimates of 13 DGVMs (see Methods for a description of TRENDY database). The 2001–2018 average based on our BLUE simulations are very similar to the TRENDY estimates for all regions (Fig. 1b). However, there are large differences between our estimates and the TRENDY estimates for the extreme values and the IAV of *S*_LAND,*B*_. We calculate the IAV as the coefficient of variation, i.e., the standard deviation of *S*_LAND,*B*_ divided by the average^16^ *S*_LAND,*B*_ between 2001 and 2018. There are some regions where the TRENDY estimates show a higher IAV of *S*_*LAND*,*B*_ than our estimates. This mainly applies to Southern Africa and especially Oceania (mostly Australia, Supplementary Fig. 2) and is probably related to the dominating effects of grasslands there (Supplementary Fig. 3), which are not captured in our BLUE results. However, ref. 27 validate the Australian carbon cycle as simulated by the TRENDY (v8) DGVMs with various observational datasets and find that the uncertainty is large (Cumulative NBP 1901-2018: -4.7 to +9.5 PgC), mainly due to different model assumptions (e.g., land cover distribution, land-use implementation, atmospheric CO_2_ concentration). Contrary to that, we estimate a much higher IAV in the boreal regions of Canada and Russia than the TRENDY models (Supplementary Fig. 4). Despite large differences between the individual models, we find that 12 out of 13 TRENDY models estimate that the IAV of the carbon sink in boreal (defined in Supplementary Fig. 5) vegetation is at least 67% (maximum: 96%) smaller than our BLUE estimates.

**Fig. 2:**
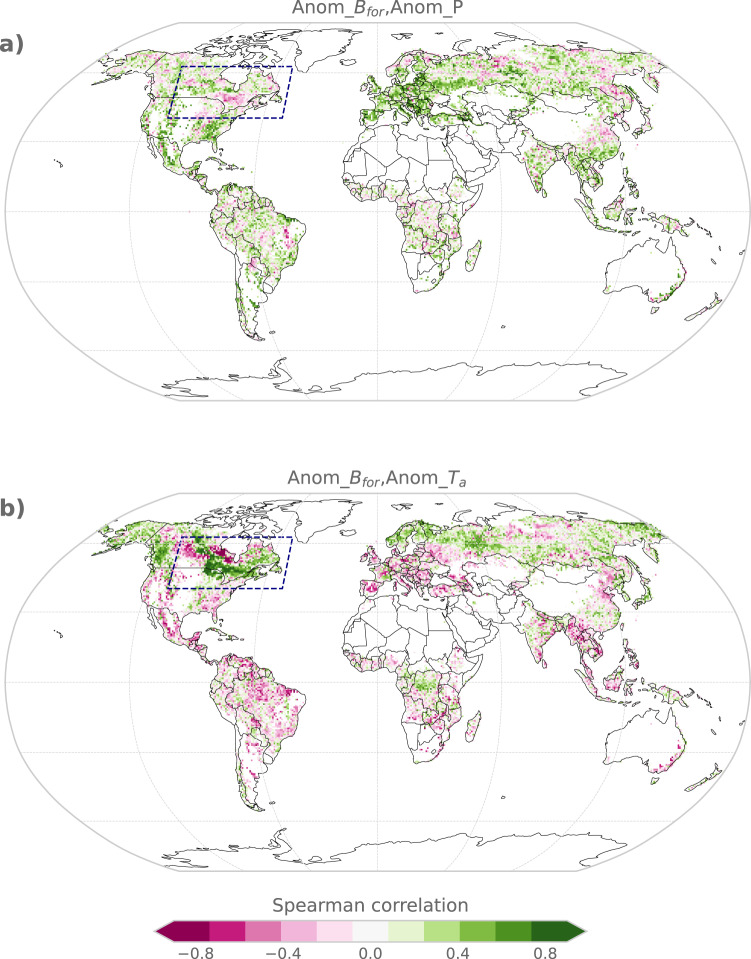
Spatial correlations between annual anomalies of climate variables and biomass carbon between 2000 and 2019. The global maps show the Spearman correlation coefficient between the time series of forest biomass carbon anomalies and the time series of **a** precipitation (P) anomalies and **b** air temperature (T_a_) anomalies. The climate variables are taken from ERA-5 reanalysis data. The anomalies are calculated by detrending each variable. The dark blue frame denotes parts of the North American boreal forest, where we find a high (>0.7) positive correlation between air temperature anomalies and biomass carbon anomalies.

**Fig. 3:**
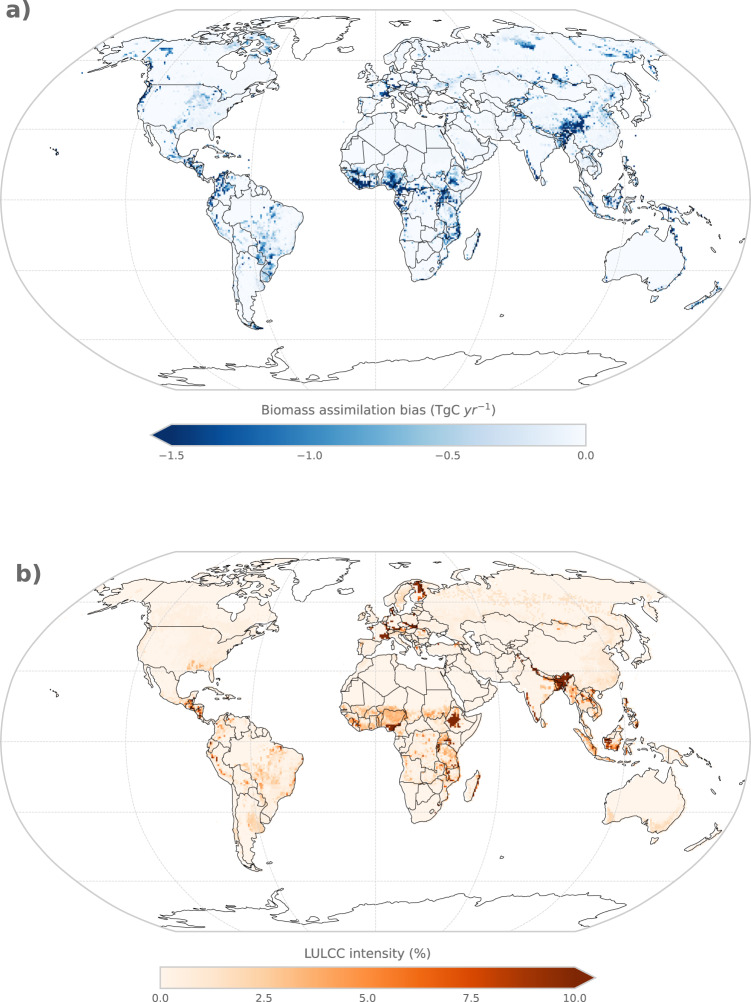
Global maps of biomass assimilation bias and LULCC intensity according to LUH2. The biomass assimilation bias (**a**) is due to uncertainties in the LULCC forcing and plant functional type distribution in BLUE and is calculated as the average (2000–2019) difference in woody biomass carbon stocks between the assimilated time series in BLUE and the observed time series by ref. 16. The LULCC intensity (**b**) is defined as the average (2000–2019) area percentage per grid cell, that is cleared or harvested. Note the regions where high biomass assimilation biases coincide with strong LULCC intensities in Northeast India, Europe, and Equatorial Africa.

The magnitude of IAV of S_LAND,*B*_ in our estimates is closely related to that from the underlying biomass changes from ref. 16. While IAV may seem high compared to regional estimates of disturbance impacts, it is not inconsistent with previous studies of land sink fluxes (see Section “Uncertainties” in the Methods Section). If we assume the IAV, as based on ref. 16, is correct, several other explanations as to why the TRENDY DGVMs estimate a lower IAV of *S*_LAND,*B*_ in boreal regions, emerge. For the North American (NAM) boreal forest, our analysis suggests that annual anomalies in air temperature have a large effect on the variability of *S*_LAND,*B*_, as estimated by BLUE. We calculated the Spearman correlation coefficient between the anomalies in annual biomass carbon and the anomalies in (1) mean annual air temperature and (2) annual precipitation sums (both from ERA-5 reanalysis data^28^). We find a strong positive correlation (mostly >0.7) between the annual mean air temperature anomaly and the annual anomaly in forest biomass carbon (Fig. 2), which mainly translates to a high IAV of *S*_LAND,*B*_, as LULCC intensity is very low in this region (Fig. 3). Our findings are similar to ref. 29, who constrain a light-use efficiency model with satellite-derived vegetation dynamics and conclude that temperature, together with water availability, strongly affects the high IAV in plant productivity in northern high latitudes with an increasing influence of temperature under global warming. We further find a spatial gradient for the temperature-vegetation carbon correlation in the NAM boreal forest, which implies decreasing (i.e., positive to negative) correlations from east to west. This gradient represents the effect of temperature-/radiation- vs. water-limited ecosystems and is supported by refs. 30, 31, who find that air temperature and radiation are the dominant factors for plant growth in the eastern parts of the NAM boreal forest, whereas precipitation and soil moisture limit plant growth in the western parts of the NAM boreal forest. Since heterotrophic respiration is also primarily temperature-limited^32^, it should be noted that increased carbon uptakes by the NAM boreal forests related to warmer years are expected to be partly offset by increased heterotrophic respiration when the total natural land sink is investigated (i.e., when natural carbon fluxes from the soil, dead wood, and litter are accounted for). The average of the TRENDY models (calculated as average biomass carbon prior to the correlation analysis) show weaker (mostly <0.5) correlations between annual anomalies in (transient) biomass carbon and temperature anomalies and do not show a switch in sign of the correlation within the NAM boreal forest from east to west, which would be in line with the spatial biomass carbon gradient shown in our results (Supplementary Fig. 6b). Despite large differences between the individual models, we find that all DGVMs show much weaker correlations than the results based on BLUE and none of the models reproduces the spatial biomass gradient (not shown). Supplementary Fig. 7 shows that the analysed correlations between air temperature anomalies and biomass carbon anomalies based on BLUE are significant (*p* < 0.05) throughout the majority of the NAM boreal forest, whereas the correlations based on the TRENDY multi-model average are only significant in some parts.

The lower IAV of *S*_LAND,*B*_ in the boreal regions based on the TRENDY results could also be related to the coarse horizontal resolution^8^, which might lead to incomplete representations of sub-grid scale processes (e.g., droughts or vegetation greening) that contribute substantially to the variability of biomass carbon. This is further supported by ref. 33, who suggest that the TRENDY (v6) models have deficits in capturing local to regional (<1000 km) spatial variability in (aboveground) biomass carbon. For reference, the NAM boreal forest area in Fig. 2 extends over a distance of around 5000 km (north-western corner to south-eastern corner of the blue frame).

Our BLUE estimates do not only suggest a larger IAV of *S*_LAND,*B*_ in some regions, but also a stronger reduction in sink capacity of the terrestrial woody vegetation in response to specific drought years in some regions. This is e.g., shown in Brazil, where our estimates indicate a 0.4–0.9 PgC yr^−1^ weaker natural carbon sink than the TRENDY multi-model average for the documented strong El Niño years in 2005^22^ and 2015^34^, and for the severe drought in the Amazon basin in 2010^22^. For 2005 and 2010, our estimates lie outside the TRENDY multi-model range. A similar dynamic is found for Europe, where our estimates suggest that the reduction in sink strength in 2015 was ten times (+0.4 PgC yr^−1^) stronger (calculated as BLUE S_LAND,B,2015_ minus TRENDY S_LAND,B,2015_) than the TRENDY multi-model average (but still within the TRENDY range) as a response to the severe drought in the same year^35^. An underestimation of the drought-mediated reduction in vegetation productivity by DGVMs is shown by other studies^36–38^ and is suggested to be partly attributable to the fact that some models do not account for heat and water stress effects on plant productivity. Another suggested influential factor is the implementation of empirically-derived response curves for temperature and moisture in most models. The response curves capture plant productivity as a function of temperature and moisture and are based on historically observed climate, which might not be accurate for unprecedented extreme events^38^. The stronger reduction in our *S*_LAND,*B*_ estimates as a response to drought events might also be related to the implicit consideration of forest degradation in our approach, which is not implemented in the DGVMs. A recent study by ref. 39 suggests that forest degradation leads to three times higher cumulative carbon losses of aboveground biomass than deforestation in the Brazilian Amazon between 2010 and 2019, with especially strong forest degradation due to el Niño events. This might be a further explanation for the differences between our results and the TRENDY results in Brazil.

## Discussion

The separation of anthropogenic and natural carbon fluxes has been identified as one of the key challenges for reconciling and integrating models and observations^14^. The approach we developed tackles this challenge by disaggregating observation-based estimates of carbon stocks into the net LULCC flux (*E*_LUC_) and the natural terrestrial sink (*S*_LAND_). Our analysis highlights the importance of observational constraints for a more realistic representation of observed global vegetation dynamics in models and the attribution of anthropogenic vs. environmental impacts.

The comparison between our BLUE simulations with transient and fixed woody biomass carbon densities suggests that the biomass carbon sinks prior to clearing and wood harvesting were larger under transient environmental conditions due to increasing atmospheric CO_2_ levels and other favorable environmental changes, which in turn led to larger carbon emissions upon clearing and wood harvesting compared to fixed environmental conditions.

**Fig. 4:**
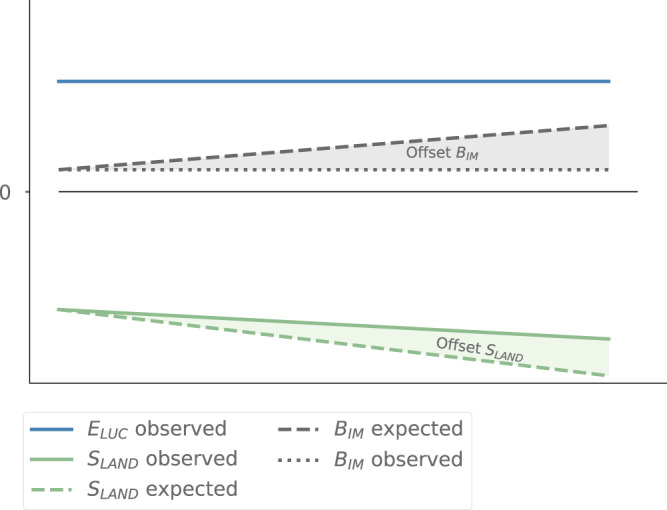
Simplified scheme of the implications of not considering synergies between environmental effects on carbon stocks and *E*_*LUC*_. Trends of each term are assumed to be linear for simplicity, which is not representative of the real dynamics. Solid lines represent how environmental effects, such as increasing CO_2_ concentrations, on each term (i.e., increase or decrease) are considered in current carbon budget approaches, such as the Global Carbon Budget (GCB)^7^. Dashed lines show the expected trends if the effect of the increasing natural terrestrial carbon sink (*S*_*LAND*_) due to environmental effects on carbon stocks were included in current approaches. Solid lines, termed “observed”, show how environmental effects on carbon fluxes from land-use and (land-use induced) land cover change activities (*E*_LUC_) and on *S*_LAND_ are considered in current approaches. The B_IM_ in current approaches is shown as dotted line (B_IM_ observed). *E*_LUC_ (*E*_LUC_ observed) is shown as a constant (excluding variability due to LULCC), because the bookkeeping models used in the GCB assume time-invariant carbon densities. Considering that the increase in *E*_LUC_ due to environmental effects is not captured and assuming that the trend of increasing *S*_LAND_ due to environmental influences is depicted accurately, B_IM_ would have to increase over time (B_IM_ expected). As this is not the case, i.e., the B_IM_ is approximately constant since 1959 (see B_IM_ observed), it is suggested that the trend of increasing *S*_LAND_ is underestimated (Offset *S*_LAND_) in the GCB assessments.

That current carbon budgeting approaches exclude the effects of environmental changes on *E*_LUC_ may have important implications for our confidence in other budget terms. The budget imbalance (B_IM_) is a measure of uncertainty in the estimated terms of the GCB, as it describes the difference between the emissions and sinks on the land, in the ocean, and in the atmosphere^7^. Following the GCB assessments, it is assumed that the atmospheric growth rate of CO_2_ (G_atm_) can be measured with high confidence, whereas the assessments of the natural carbon sinks on land and in the ocean are more uncertain^7^. If the *S*_LAND_ trends were depicted accurately, there should be an increase in *S*_LAND_ when considering environmental effects on carbon stocks (mainly due to more favourable growing conditions under elevated CO_2_ levels), while the quantification of *E*_LUC_ through BKMs excludes all environmental effects, i.e., the budget imbalance would have to increase over time. Since the budget imbalance has been approximately constant with no trend since 1959 in the GCB assessments, we conclude that the global trend of increasing *S*_LAND_ is not captured accurately (Fig. 4) in the GCB.

Our analysis highlights the potential of using observational constraints for improving and reconciling model estimates, as multi-model uncertainties in *E*_LUC_ estimates are reduced to a substantial degree by assimilating observation-based woody biomass carbon densities in a BKM. Further, our results suggest that state-of-the-art DGVMs have deficits in capturing the IAV of *S*_LAND,*B*_ and the response of terrestrial vegetation to extreme events. These findings are in line with other recent studies, which find several explanations for the limitations of DGVMs to represent observed patterns in terrestrial carbon cycle dynamics. Our model-data integration also reveals hotspots where model assumptions or the underlying LULCC forcing are inconsistent with the observed carbon dynamics (see paragraph on “Uncertainties” in Methods).

A major advantage of our framework is that it can be extended flexibly to updated datasets and can constantly be improved with more observational datasets being made available.

There are various data additions to our approach that we would consider to be especially valuable for improving model-data assimilated estimates of global terrestrial carbon cycle dynamics. First, the inclusion of a time series of observational estimates of carbon contained in non-woody vegetation and soils would be a very valuable addition to our approach, as recent studies suggest a major contribution of soils to the IAV of the terrestrial carbon sink in transitional regimes^26,40^. Second, extending the time series of carbon stocks to sub-annual time scales would enable a more detailed analysis of the intra-annual response of the terrestrial carbon cycle to extreme events, which has been shown to be highly variable in space and time^34^. The need for observation-based estimates of terrestrial carbon stocks that are consistent in space and time is acknowledged by a wider community and steps towards this goal are currently being undertaken through various recently launched (Global Ecosystem Dynamics Investigation, ECOsystem Spaceborne Thermal Radiometer Experiment on Space Station)^41,42^ and upcoming satellite missions (e.g., Geostationary carbon cycle observatory, BIOMASS)^11,43^, which are dedicated to measuring the Earth’s vegetation properties. Combining those measurements with ground-based observations and models will be a major contribution towards more reliable estimates of environmental and anthropogenic CO_2_ fluxes, which are independent of national GHG inventories. This is crucial for monitoring country-level emission commitments according to the 2015 Paris agreement and for future climate change mitigation and adaptation strategies.

## Methods

### External datasets

#### Woody biomass carbon data

The dataset by ref. 16 maps annual global woody biomass carbon densities for 2000–2019 at a spatial resolution of ~10 km. The annual estimates represent averages for the tropical regions and growing-season (April–October) averages for the extra-tropical regions. Ref. 16 analyse global trends of gains and losses in woody biomass carbon for 2000–2019. Overall, they find that grid cells with (significant) net gains of vegetation carbon are by a factor of 1.4 more abundant than grid cells with net losses of vegetation carbon, indicating that there is a global greening trend when only considering the areal extent of biomass gains and not the magnitude of carbon gains. Their regionally distinct analysis of trends shows that almost all regions, except for the tropical moist forests in South America and parts of Southeast Asia, experienced net gains in biomass carbon. On the country scale, the largest net increase in biomass carbon is shown in China, which is mainly attributed to the large-scale afforestation programs in the southern part of the country and increased carbon uptake of established forests. On the other hand, the largest vegetation carbon losses are shown for Brazil and Indonesia, which is partly attributed to deforestation, degradation, and drought events. All of the mentioned trends have been found to be significant^16^. The decreasing carbon sink in Brazil is in line with ref. 44, who, considering both natural and anthropogenic fluxes, show that the southeastern Amazon has even turned from a carbon sink to a carbon source, mainly owing to fire emissions from forest clearing. Isolating carbon fluxes in intact, old-growth Amazonian rainforests (i.e., *S*_LAND,*B*_), ref. 45 also find evidence for a significantly decreasing carbon sink due to the negative effects of increasing temperatures and droughts on carbon uptake since the 1990s.

The dataset was remapped to the BLUE resolution of 0.25^∘^ through conservative remapping (i.e., area-weighted averaging).

#### ERA-5 data

The ERA-5 variables were downloaded from the Copernicus Climate Data Store (https://cds.climate.copernicus.eu/cdsapp#!/home). Monthly air temperature (T_a_) at 2 m height was averaged over each year, and annual precipitation was calculated by taking the sum of the monthly total precipitation (P). Both variables were regridded from the original resolution of ~0.1° to 0.25° resp. to the TRENDY resolution of 0.5° through conservative remapping.

#### TRENDY data

We used the TRENDY model ensemble version 8 (conducted for the 2019 GCB; ref. 8). We used net biome production (NBP) and annual vegetation carbon stocks (cVeg) for 2000–2018 from four different model setups (S2, S3, S5, and S6) and eight resp. 13 DGVMs (depending on the data available). The selection of DGVMs is done as in ref. 19 (Supplementary Tab. 3), but we included one additional model (ISAM) for the S2 simulations. The terrestrial biomass carbon sink (*S*_LAND,*B*_) was calculated for 13 DGVMs following the GCB 2020 approach, i.e., from the S2 simulation, which is the simulation without LULCC (i.e., fixed pre-industrial land cover) under transient environmental conditions (climate, nitrogen deposition, CO_2_ evolution). *S*_LAND,*B*_ is the annual difference of cVeg and makes no statements about the further fate of biomass if cVeg decreases. *S*_LAND,*B*_, therefore, should not be interpreted as equivalent to the flux to/from the atmosphere, since parts of cVeg may be transferred to litter, dead wood, or soil. The same applies to our BLUE estimates of *S*_LAND,*B*_, ensuring comparability between our BLUE estimates and the TRENDY estimates. Increases (decreases) of cVeg between two years are a net uptake (release) of carbon from the terrestrial biosphere. The global sums of biomass carbon stocks under transient climate and CO_2_ were calculated from the S3 setup (LULCC under historical environmental conditions), whereas the S5 setup provides biomass carbon under constant present-day environmental forcing (closest to the classical bookkeeping approach). In line with the GCB, *E*_LUC_ was calculated under historical environmental conditions as the difference in NBP between the S2 and S3 simulations (*E*_LUC_ = NBP_S2 - NBP_S3). *E*_LUC_ under constant present-day environmental forcing was calculated as the difference in NBP between the S6 (fixed pre-industrial land cover under present-day environmental forcing) and S5 simulations (*E*_LUC_ = NBP_S6 - NBP_S5)^19^. All datasets were remapped to a common resolution of 0.5^∘^ through conservative remapping (area-weighted average) for the data analysis.

### Assimilation of observed woody biomass carbon in BLUE

The observed woody biomass carbon densities by ref. 16 are assimilated in BLUE in several steps.

#### Carbon transfer in the default setup of BLUE

The BLUE simulation is started in AD 850. Biomass and soil vegetation carbon densities are based on ref. 17 (see ref. 5 for details). These carbon densities are specific for eleven natural PFTs (Supplementary Fig. 3), which are assigned one of four land cover types (primary land, secondary land, cropland or pasture). The LULCC forcing is based on the LUH2 dataset^18^, defining the vegetated fractional area of each grid cell that is affected by a land-use transition. Each transition may lead to a change from one land cover type (=source land cover type: *j*) to another land cover type (=target land cover type: $j^{\prime}$). In the case of wood harvesting on secondary land, $j=j^{\prime}$, whereas all other transition types (e.g., clearing for agricultural expansion, abandonment of agricultural lands) induce a change in land cover. The fractional grid cell areas undergoing transitions are further distributed across PFTs proportionally to the temporally constant PFT area fractions (Supplementary Fig. 3). Upon each land-use transition, biomass carbon is transferred between the source land cover type and the target land cover type, whereby the amount of transferred carbon depends on the biomass carbon density of the source and target land cover types (in the respective PFT) and the area affected by the transition (in the respective PFT). The temporal evolution of the biomass carbon pool after any type of land-use transition is approximated by an exponential function with different time constants for decay and regrowth, depending on the type of land-use transition. The time constants are based on linear estimates by ref. 17, which are converted to exponential time constants. A detailed explanation of the exponential model can be found in ref. 5.

While in the default setup, changes are only due to LULCC, our assimilation approach now introduces environmental effects on woody vegetation carbon by assimilating the observed woody biomass carbon densities in BLUE from 2000 onward according to the methodological considerations explained below.

#### Calculation of woody biomass carbon densities for different land cover types and PFTs

Within each 0.25° cell of the global grid, the (remapped) woody biomass carbon density from ref. 16 must be the sum of woody biomass carbon stored in all woody PFTs of all woody land cover types. The distribution of the woody biomass carbon across PFTs and land cover types is achieved by distributing the observed (i.e., actual) woody biomass carbon densities (*ρ*_*Ba*_) from ref. 16 across the two land cover types (*j*) and the eight PFTs (*l*) that can be woody vegetation (primary land, called virgin, “v” in BLUE and secondary, “s”, land) according to the fraction of total woody biomass carbon (*f*_*B*_) contained in each land cover type and each PFT (*f*_*B*,*j*,*l*_) as estimated by BLUE. *f*_*B*,*j*,*l*_ varies for different PFTs and land cover types, depending on their history of LULCC and their potential for carbon uptake (i.e., the potential carbon densities).

*f*_*B*,*j*,*l*_ is extracted from the default simulations for the first year of the time series (i.e., 2000) and calculated for subsequent years from the BLUE simulations using the assimilated woody vegetation carbon densities for that year:

1 $${f}_{B,j,l}(t)=\frac{{C}_{B,j,l}(t)}{{C}_{B}(t)}$$

where *C*_*B*_ is the woody biomass carbon stock.

Consequently, the assimilated woody biomass carbon stock per cover type and PFT (C_B_as,j,l_) at each time step can be calculated as:

2 $${C}_{B\_as,j,l}(t)={\rho }_{Ba}(t)\;*\;A\;*\;{f}_{B,j,l}(t)$$

with *j*{*v*, *s*}; *l*{1. . 8}; *t*{2000. . 2019}. *A* is the area per grid cell.

#### Thresholds for excluding inconsistent woody biomass carbon densities

We eliminate unrealistically large values for woody biomass carbon densities that our assimilation framework produces. Woody biomass carbon densities in BLUE that exceed the highest value (~374 t ha^−1^) of the original dataset indicate inconsistencies between the observed woody biomass carbon estimates and the fractional grid cell areas per PFT and land cover types that BLUE simulates. To account for uncertainties related to the criteria for exclusion of grid cells, multiple threshold approaches are applied and the results are compared. To maintain a temporally and spatially consistent time series of woody biomass carbon, grid cells that are excluded according to the chosen threshold approach are interpolated through linear barycentric interpolation. A first approach relies on a uniform upper threshold of <375 t ha^−1^ for woody biomass carbon densities. This approach leads to the exclusion of ~3% of all grid cells, but is considered conservative in the sense that it may lead to an overestimation of woody biomass carbon densities of non-forested land, since it is expected that the maximum value of ~374 t ha^−1^ occurs in heavily forested grid cells only. To account for this potential overestimation, additional threshold approaches are applied by cutting the distribution of grid cells with woody biomass carbon densities smaller than 375 t ha^−1^ to a range of specific percentiles and choosing the values corresponding to each percentile as upper thresholds for the exclusion of further grid cells. In the first step, we choose the 97th, 98th, and 99th percentiles and evaluate the resulting dynamics of total vegetation carbon in terms of their agreement with the original dataset by ref. 16. The evaluation is done by analyzing the results from each percentile threshold approach in terms of the global dynamics of the biomass carbon stocks in comparison to the estimates from ref. 16 (see Supplementary material). This analysis reveals that the annual dynamics (i.e., increase/decrease) of the woody biomass carbon stocks start to diverge strongly from the original time series for thresholds smaller than the 99th percentile. This is related to an enhanced loss of spatial and temporal variability of the assimilated biomass carbon stocks due to an increased number of interpolated grid cells with smaller percentile thresholds. Consequently, we choose the two approaches with (1) <375 t ha^−1^ and (2) <99th percentile of 375 t ha^−1^ as upper limits for the exclusion of inconsistent biomass carbon densities and use their average, unless indicated otherwise. Both threshold approaches are applied to each woody PFT and the two woody land cover types separately over the whole time series (2000–2019). Consequently, it is ensured that differences in carbon storage potential between different PFTs and land cover types are considered within the percentile threshold approach.

#### Model initialization

In our transient woody biomass carbon approach, we need to initialize the woody biomass pools in BLUE at each time step (i.e., each year) to account for changes in biomass carbon densities due to environmental processes. As we do not assimilate soil carbon densities in our approach, the soil carbon pools are initialized once at the beginning of the BLUE simulations (described below) and subsequently only altered by LULCC. The re-initialization for the woody biomass pools at each time step is necessary, as BLUE only explicitly simulates annual changes in biomass carbon densities due to LULCC. In the default approach, total biomass carbon is partitioned between equilibrium pools (${\bar{C}}_{B,j,k,l}$) and excess pools (*δ*_*B*,*j*,*k*,*l*_). The former mark the carbon stock that the biomass pools strive to reach (i.e., the carbon stock that the PFT and land cover type would reach after a sufficiently long time after a land-use disturbance), while the latter indicate whether the current biomass carbon stock is in equilibrium (*δ*_*B*,*j*,*k*,*l*_ = 0) or in excess of equilibrium (*δ*_*B*,*j*,*k*,*l*_ ≠ 0) (Note that *k* is the (land-use) history type, including clearing (“l”), harvest (“h”), abandonment (“a”), other (“g”)). An in-depth explanation of the different pool types in BLUE can be found in the original documentation by ref. 5. Biomass carbon is assumed to be in equilibrium upon model initialization, i.e., the equilibrium pools contain all biomass carbon and the excess pools are zero. Upon each land-use transition, the equilibrium and excess biomass carbon pools are altered, depending on the transition type.

In our approach, the model initialization is done by distributing the assimilated woody biomass carbon among the equilibrium biomass pools for all woody PFTs and all land cover types (see Supplementary materials for the handling of non-woody land cover types). This means that the equilibrium biomass pools and all excess biomass pools are then re-initialized at each time step (annually) of the simulation from 2000 onward. The excess carbon pools are changed upon each land-use transition, whereby the spatially explicit actual woody biomass carbon densities derived from ref. 16 replace the woody biomass carbon densities based on ref. 17 from 2000 onward. The actual woody biomass carbon densities from ref. 16 are assimilated in BLUE at the beginning of each year X and subsequently altered by the land-use transitions in year X. Consequently, the BLUE output of carbon stocks for year X represents the end of year X and changes in carbon stocks between year X+1 and year X are attributed to year X+1. Legacy fluxes (i.e., carbon fluxes from land-use that do not occur in the same time step as the corresponding land-use event) are tracked according to the approach explained below.

#### Handling of legacy carbon fluxes

Due to repeated initialization of the (equilibrium and excess) biomass carbon pools at each time step, legacy fluxes are not accounted for and need to be tracked separately. Such legacy fluxes from/to the atmosphere to/from the terrestrial woody biomass occur due to LULCC prior to the current time step, e.g., because the forest regrows slowly or because cleared biomass decomposes slowly on site or in products. We track these legacy fluxes separately for those from the LULCC prior to the assimilation period (2000–2019), and those occurring during the assimilation period, which are caused by the LULCC transitions and other biomass changes. To track the former, we introduce an additional set of excess pools *δ*_*B*,leg<2000_ that include all excess woody biomass carbon from land-use transitions prior to 2000 upon initialization of the actual woody biomass carbon pools in 2000. Legacy carbon fluxes from land-use transitions prior to 2000 (*θ*_*B*,leg<2000,*j*,*k*,*l*_) are calculated as in the default approach (Note: carbon fluxes from LULCC in time step (t) from/to the land to/from the atmosphere are realized at the beginning of time step (t+1) in BLUE):

3 $${\theta }_{B,leg\,{ < }\,2000,j,k,l}(t)={\delta }_{B,leg\,{ < }\,2000,j,k,l}(t-1)-{\delta }_{B,leg\,{ < }\,2000,j,k,l}(t-1)\;*\;{{{{{{{{\rm{e}}}}}}}}}^{\frac{-1}{{\tau }_{{{{{{{{\rm{B}}}}}}}},{{{{{{{\rm{j}}}}}}}},{{{{{{{\rm{k}}}}}}}},{{{{{{{\rm{l}}}}}}}}}}}$$

with *j*{*v*, *s*, *p*, *c*}; *l*{1. . 8}; *t*{2000. . 2019}; *k*{*l,h,a,g*}. *τ* is the time constant for relaxation processes, which varies for different pool types (biomass or soil), land cover types, and PFTs.

Excess woody biomass carbon from transitions from 2000 onward is tracked in another set of separate pools (*δ*_*B,leg*≥2000_) to account for ≥2000 legacy fluxes. *δ*_*B,leg*≥2000_ is adjusted at the beginning of each time step for all excess woody biomass carbon from the previous time step minus fluxes to/from the atmosphere (Eq. (4a)) (*θ*_*B,leg*≥2000,*j*,*k*,*l*_) from relaxation processes in the respective time step (Eq. (4b)):

4a $${\delta }_{B,leg\ge 2000,j,k,l}(t)={\delta }_{B,leg\ge 2000,j,k,l}(t-1)+{\delta }_{B,j,k,l}(t-1)-{\theta }_{B,leg\ge 2000,j,k,l}(t)$$

4b $${\theta }_{B,leg\ge 2000,j,k,l}(t)={\delta }_{B,leg\ge 2000,j,k,l}(t-1)-{\delta }_{B,leg\ge 2000,j,k,l}(t-1)\;*\;{{{{{{{\rm{{e}}}}}}}^{\frac{-1}{{\tau }_{B,j,k,l}}}}}$$

Carbon fluxes between the terrestrial woody biomass pool and the atmosphere pool at each time step, including all legacy fluxes (*θ*_*B*,*j*,*k*,*l*_), can then be calculated as the sum of instantaneous carbon fluxes at the current time step (resulting from LULCC in the previous time step), legacy carbon fluxes prior to 2000 and legacy carbon fluxes from 2000 onward, but prior to the current time step (resulting from LULCC prior to the previous time step).

5 $${\theta }_{B,j,k,l}(t)=	{\delta }_{B,j,k,l}(t-1)-{\delta }_{B,j,k,l}(t-1)\;*\;{{{{{{{{\rm{e}}}}}}}}}^{\frac{-1}{{\tau }_{{{{{{{{\rm{B}}}}}}}},{{{{{{{\rm{j}}}}}}}},{{{{{{{\rm{k}}}}}}}},{{{{{{{\rm{l}}}}}}}}}}}\\ 	+ {\theta }_{B,leg\,{ < }\,2000,j,k,l}(t)+{\theta }_{B,leg\ge 2000,j,k,l}(t)$$

#### Derivation of the terrestrial woody biomass carbon sink

To isolate anthropogenic from environmental (=*S*_LAND,*B*_) carbon fluxes from woody vegetation, we performed two simulation setups based on different approaches for assimilating the observed woody biomass carbon densities. The biomass estimate by ref. 16 includes carbon stored in living woody vegetation (trees and shrubs), whereas carbon stored in dead plant material (litter, harvested wood products) is not included in the estimate. Consequently, the change in woody biomass carbon stocks within a certain time step results from carbon sources and sinks driven by LULCC in the respective time step, from carbon sinks due to regrowth of vegetation driven by past LULCC (i.e., prior to the respective time step) and from all environmental processes (on managed and unmanaged lands) in the respective time step:

6 $${{\Delta }}C(t)={{\Delta }}{C}_{{{{{{\mathrm{source}}}}}}}(t)+{{\Delta }}{C}_{{{{{{\mathrm{sink}}}}}}}(t)+{{\Delta }}{C}_{{{{{{\mathrm{reg}}}}}}\_{{{{{\mathrm{leg}}}}}}}(t)+{{\Delta }}{C}_{{{{{{\mathrm{source}}}}}},{{{{{\mathrm{env}}}}}}}(t)+{{\Delta }}{C}_{{{{{{\mathrm{sink}}}}}},{{{{{\mathrm{env}}}}}}}(t)$$

where Δ*C*_source_ resp. Δ*C*_sink_ are sources resp. sinks of biomass carbon due to LULCC in the current time step, Δ*C*_reg_leg_ are sinks of biomass carbon due to regrowth of vegetation from LULCC prior to the current time step and Δ*C*_source,env_ resp. Δ*C*_sink,env_ are sources resp. sinks of biomass carbon due to environmental processes in the current time step. We performed additional BLUE simulations with fixed (i.e., stationary in time) woody biomass carbon densities to split the carbon fluxes from woody vegetation into the anthropogenic and environmental terms of Eq. (6). The fixed woody biomass carbon setup is based on the 2000 estimates derived from ref. 16. As in the transient simulations, model initialization is done from the same state of woody biomass carbon in 2000 (i.e., anthropogenic and environmental effects on woody biomass carbon prior to 2000 are implicitly captured), but changes in woody biomass carbon in the subsequent years are only driven by LULCC in the fixed setup. In the fixed woody biomass carbon simulations, there is no need for separately tracking legacy fluxes from 2000 onward, since the biomass carbon pools are only initialized in 2000 and the excess pools are altered subsequently without re-initialization. Legacy carbon fluxes from land-use transitions prior to 2000 are considered following the same approach as in the transient woody biomass carbon density setup. The terms of Eq. (6) that capture environmental changes in biomass carbon stocks are only included in the BLUE simulations with transient biomass carbon, whereas biomass carbon changes driven by land-use change are captured in both the transient and fixed biomass carbon simulations. Consequently, our BLUE simulations allow us to isolate all environmental effects on woody biomass carbon by taking the difference in woody biomass carbon stocks between the two BLUE simulation setups:

7 $${S}_{{{{{{\mathrm{LAND}}}}}},B}(t)={{\Delta }}{C}_{{{{{{\mathrm{trans}}}}}},B}(t)-{{\Delta }}{C}_{{{{{{\mathrm{fix}}}}}},B}(t)$$

This term, the natural carbon sink in terrestrial woody vegetation, represents the net effect of environmental processes on managed and unmanaged lands on the terrestrial woody living vegetation.

#### Uncertainties

The main sources of uncertainty that affect the results from our biomass assimilation approach are (1) model assumptions regarding the global LULCC dynamics and the rates of vegetation regrowth, (2) potential misattributions of anthropogenic fluxes as natural fluxes owing to incomplete data on LULCC, and (3) uncertainties within the original time series of woody biomass carbon densities by ref. 16. We analyse the different sources of uncertainty as described in the following.

(1) The difference between the observed woody vegetation carbon stocks from ref. 16 and the woody vegetation carbon stocks at the beginning of each time step in the transient BLUE setup (=“assimilated woody biomass carbon”) can be used to evaluate the LULCC forcing and the PFT distribution in BLUE. Since the observed and the assimilated woody vegetation carbon time series are not independent of each other, the comparison solely aims at identifying potential model uncertainties. The observed woody vegetation carbon densities are assimilated into BLUE at each time step according to the spatial distribution of the land cover types and PFTs (see Eq. (2)). Consequently, a larger difference between the observed woody vegetation carbon stocks and the assimilated woody vegetation carbon stocks would indicate that the actual LULCC dynamics and/or the spatial distribution of PFTs are not captured well in BLUE. We call this difference the “biomass assimilation bias”. The average global biomass assimilation bias (±1 SD) amounts to 29 ± 4 PgC between 2000 and 2019. The agreement between the observational dataset by ref. 16 and the assimilated woody biomass carbon in terms of the trends in global biomass carbon is quantified as the number of years that show the same trend in both datasets related to the previous year divided by the total number of years. Following this definition, a temporal agreement of 100% would mean that the observed dataset and the assimilated dataset show the same trend in biomass carbon for all years. The regionally averaged agreement in the estimated biomass carbon trends is generally high (>80%) (Supplementary Fig. 8) but smaller in regions with strong LULCC dynamics (tropics and Europe). Some local hotspots exist (Fig. 3), where differences between the observed dataset and the assimilated dataset are larger. These are mainly located in South- and Southeast Asia, Europe, and Equatorial Africa (Fig. 3a), where clearing and wood harvesting rates of the forest as prescribed in the LULCC forcing (Fig. 3b) are very high, leading to much lower biomass carbon estimates in our assimilated woody biomass carbon estimates than in the observed time series. This suggests that the clearing and/or wood harvesting rates are overestimated in the LULCC forcing and/or the rate of vegetation regrowth is underestimated in BLUE, leading to a high biomass assimilation bias for the mentioned regions, which further affects *E*_LUC_ (see Results).

We further assessed the validity of our *S*_LAND,*B*_ estimates in terms of the high IAV shown in Canada, Russia, Brazil, and Europe. The comparison of our time series of assimilated woody biomass carbon to the original time series by ref. 16 shows that our assimilated dataset is very close to the original dataset in the respective regions and that the high IAV is also shown in the original time series^16^. Consequently, we conclude that the high IAV is not introduced by uncertainties in our model-data integration. Nevertheless, we acknowledge that the estimated IAV in NAM (especially Canada) may seem high compared to the IAV of carbon fluxes due to natural disturbances estimated by the National Inventory Report of Canada^46^ or to specific disturbance events, such as the mountain pine beetle outbreak in British Columbia in the early 2000s^47^. However, our estimated IAV of S_LAND,*B*_ of up to 2 PgC yr^−1^ in NAM is not inconsistent with previous studies estimating annual changes of the total land sink^48^ resp. the natural land sink^49,50^ of up to 3 PgC yr^−1^, including a switch in sign of the flux. Furthermore, ref. 51 combine atmospheric CO_2_ measurements with inverse modelling and show that the North American net ecosystem exchange (NEE) between 2007 and 2015 and its variability was strongly driven by el Niño (more than average carbon uptake) and la Niña (less than average carbon uptake) conditions, with monthly anomalies (related to the mean for 2007–2015) of up to ±1.5 PgC yr^−1^. They further find that the boreal coniferous forest is among the ecosystems with the largest difference in NEE anomalies between el Niño and la Niña periods. Our S_LAND,*B*_ estimates broadly follow the dynamics described by ref. 51, with higher than average carbon uptakes during el Niño conditions in 2010 and 2015 and lower than average carbon uptakes resp. carbon releases during la Niña conditions in 2011. Furthermore, we wish to clarify that we assume that the magnitude of fluxes to/from the atmosphere from/to the biomass is smaller than our estimated changes in S_LAND,*B*_, since we include depositions to dead matter and soil in our estimates. According to the global carbon budget decomposition by ref. 49, around 50% of annual gross anthropogenic emissions (including natural fluxes on managed lands, as described in ref. 52) between 2000 and 2015 was due to direct emissions (i.e. in the year of the respective disturbance), while the rest was attributable to legacy fluxes (onsite decomposition and wood product degradation). Assuming a similar dynamic for (solely) natural disturbances, we expect that the magnitude of annual carbon fluxes to/from the atmosphere from/to the biosphere is lower - depending on the degree and type of disturbance - than the annual changes in S_LAND,*B*_. The mentioned considerations highlight the need for future, independent estimates – especially on a regional scale – to foster our understanding of the IAV of terrestrial carbon fluxes. Furthermore, ref. 16 compared their estimated IAV of woody biomass carbon with FAO estimates of forest carbon, showing that there is no systematic overestimation of IAV in the mentioned regions.

(2) A general shortcoming of all accounting approaches based on observational datasets is their inability to capture all anthropogenic activities related to LULCC. Consequently, there might be anthropogenic carbon fluxes that are classified as environmental fluxes in our approach, simply because the LULCC forcing is not capturing the underlying anthropogenic activities completely. This caveat applies foremost to certain types of anthropogenic degradation: while the LULCC forcing covers logging (implemented as wood harvesting in BLUE) and rangeland degradation, it does not account for degradation caused by anthropogenic fires, which might lead to misattributions of the related fluxes towards *S*_LAND,*B*_. However, there is currently no (global) dataset available that separates anthropogenic and natural degradation fires and that would allow us to provide an uncertainty estimate for the misattributed fluxes.

(3) Reference 16 defines errors in the order of ±0.5% (2 PgC) related to the 2000–2019 average global sum of carbon contained in woody vegetation (381 PgC). The error estimate includes pixel-level uncertainty and modeling uncertainty from parameter estimation. The global uncertainty range of ±0.5% is considered in all of our aggregated global estimates of woody biomass carbon. This means that in Table 1, the uncertainty range of ±2 PgC for the global living biomass (i.e., woody plus herbaceous vegetation) carbon stocks refers to the woody vegetation estimate only (357 PgC).

Global maps of absolute and relative pixel-level uncertainty (Supplementary Fig. 9) are provided and can be used as a reference to evaluate the accuracy of our estimates for different regions.

## Supplementary information

Supplementary Information

## Data Availability

The *E*_LUC_ data, *S*_LAND,*B*_ data, correlations between biomass carbon anomalies and climate anomalies, and the uncertainty data generated in this study have been deposited in World Data Centre for Climate (WDCC) database provided by the German Climate Computing Center (DKRZ: Deutsches Klimarechenzentrum GmbH) under 10.26050/WDCC/MoDataInToTr21stCLandFl. The LUH2 data were available at https://luh.umd.edu/data.shtml. The woody biomass carbon time series by ref. 16 is available at 10.5281/zenodo.4161694. The TRENDY v8 ensemble of simulation outputs is available upon request at https://sites.exeter.ac.uk/trendy.

## Code availability

The code for the analysis in this paper is available on request to the corresponding author.
