# Supplementary material for: Tracking 21st century anthropogenic and natural carbon fluxes through model-data integration
Source: Nat Commun. 2022 Sep 26;13:5516. doi: 10.1038/s41467-022-32456-0 (PMC9512848; doi:10.1038/s41467-022-32456-0)
Supplement: Supplementary file 1 — Supplementary Information [file 41467_2022_32456_MOESM1_ESM.pdf]

Supplementary materials: Tracking 21st century anthropogenic and natural carbon fluxes through model-data integration

Selma Bultan*[1], Julia E.M.S. Nabel[2,3], Kerstin Hartung[a1], Raphael Ganzenmüller[1], Liang Xu[b4], Sassan Saatchi[4], and Julia Pongratz[1,2]

[1] *Department of Geography, Ludwig-Maximilians-Universität, Munich, Germany*
[2] *Max Planck Institute for Meteorology, Hamburg, Germany*
[3] *Max Planck Institute for Biogeochemistry, Jena, Germany*
[4] *Jet Propulsion Laboratory, California Institute of Technology, Pasadena, CA, USA*
[a] *Now at: Deutsches Zentrum für Luft- und Raumfahrt, Institut für Physik der Atmosphäre, Oberpfaffenhofen, Germany*
[b] *Now at: Pachama Inc., San Francisco, CA, USA*

***Correspondence: selma.bultan@lmu.de**

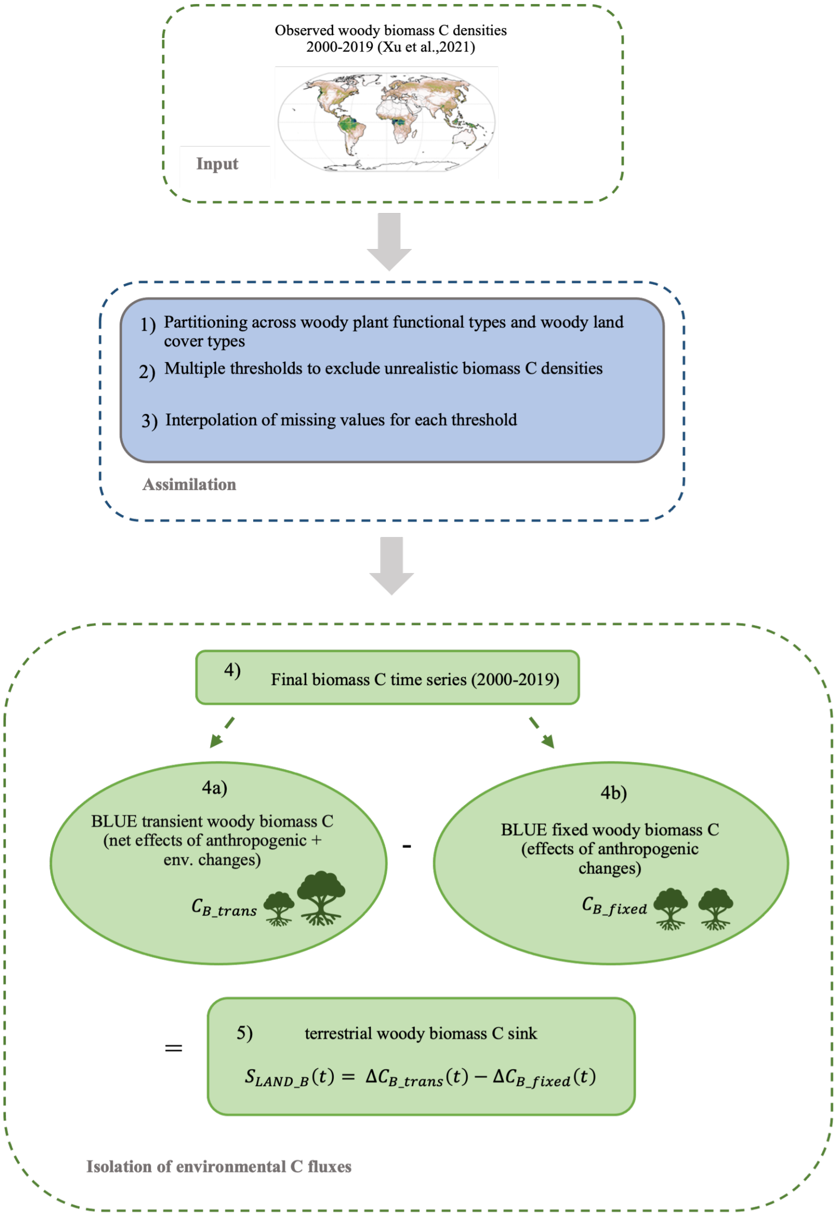

Supplementary Figure 1: Schematic overview of the model-data assimilation framework used to isolate environmental terrestrial carbon sources and sinks (=S_{LAND}). The individual steps (1-5) are explained in Section 2. Pre- and post-processing steps are shown by filled squared boxes. Filled circles represent the model simulations.

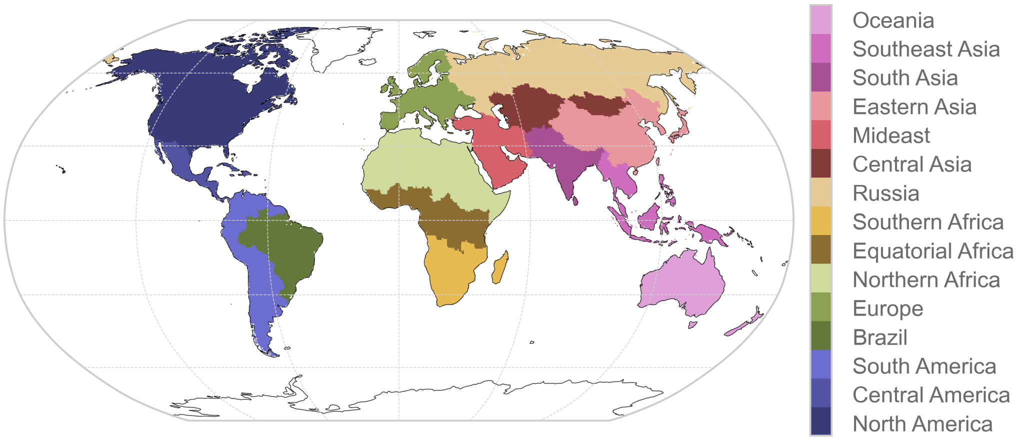

Supplementary Figure 2: Spatial distribution of the 15 regions used for aggregating the model results. The region definitions are based on the RECCAP-2 project, but we further aggregated Northern South America and Southwest South America to South America and aggregated China, Korea and Japan as Eastern Asia.

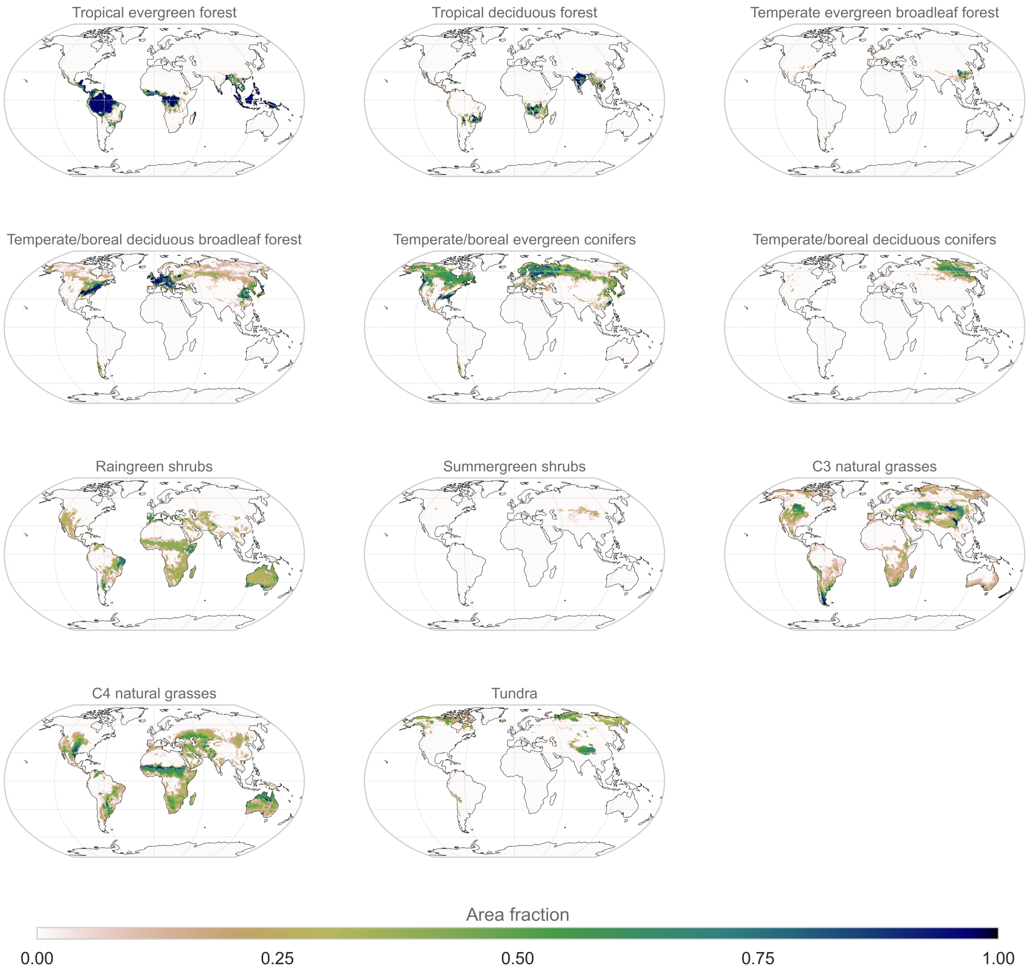

Supplementary Figure 3: Fractional grid cell area covered by each of the eleven natural vegetation types ("Plant Functional Type") in BLUE.

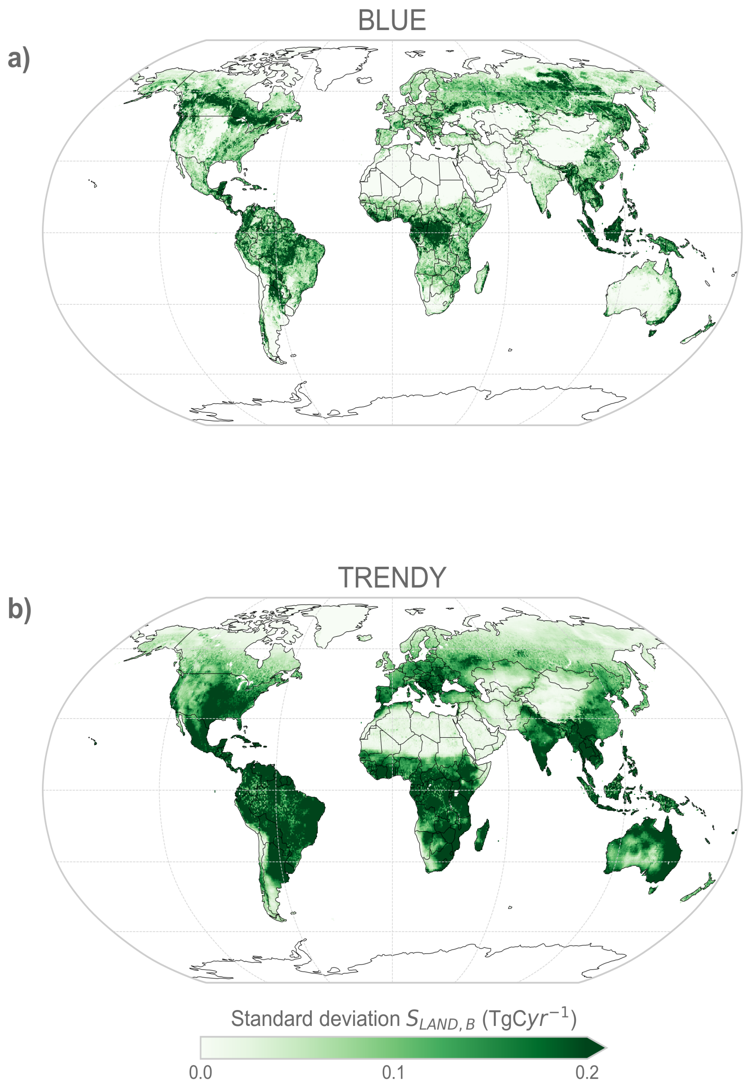

Supplementary Figure 4: Spatial comparison of the interannual variability of $S_{LAND,B}$ between BLUE (a) (woody vegetation) and the average of 13 TRENDY DGVMs (b) (all vegetation types). The interannual variability is calculated as standard the deviation between 2000 and 2018 to emphasize areas with high absolute values of $S_{LAND,B}$ and a large interannual variability.

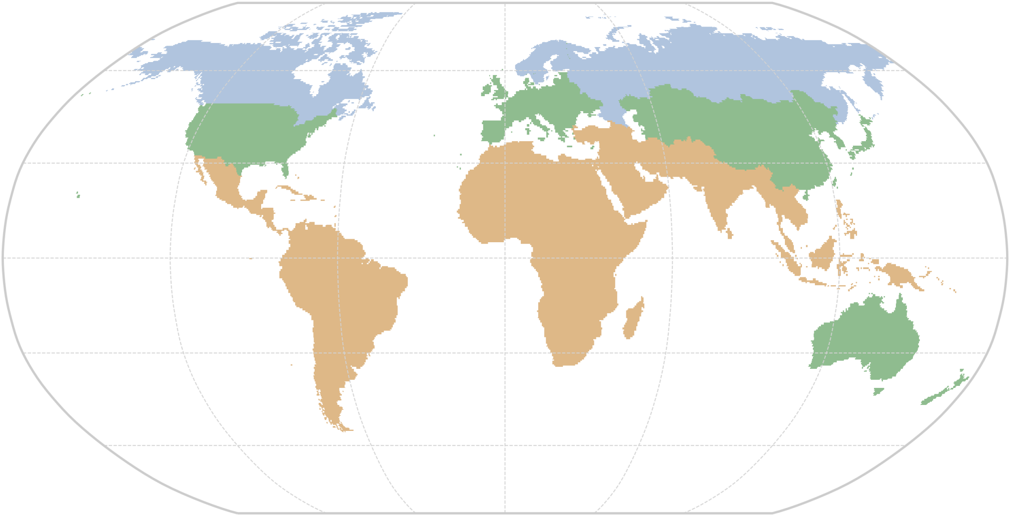

Supplementary Figure 5: Global map of different biomes. The biomes were used for aggregating the forest biomass carbon stocks (Tab. 2). The boreal biome is visualized in blue, the temperate biome is shown in green and the tropical biome is shown in light brown. The biome mask is based on the RECCAP-2 regions map (Supplementary Fig. 2), which is further aggregated according to the definitions by [1].

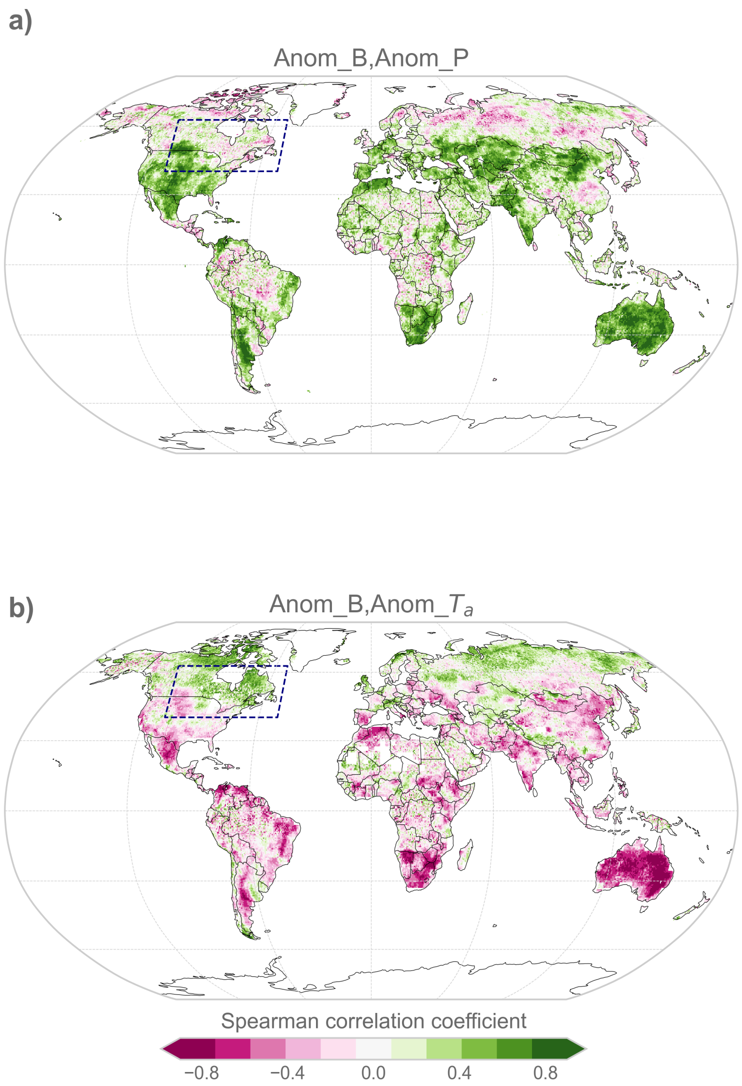

Supplementary Figure 6: Spatial correlation between annual anomalies of climate variables and biomass carbon. The global maps show the Spearman correlation coefficient between the time series of biomass carbon anomalies (as opposed to woody biomass carbon anomalies in Fig. 2) as estimated by averaging over eight TRENDY DGVMs (prior to the correlation analysis for the S3 setup) and the time series of a) precipitation (P) anomalies and b) air temperature (Ta) anomalies. The climate variables are taken from ERA-5 reanalysis data. The anomalies are calculated by detrending each variable. The dark blue frame denotes parts of the North American boreal forest, where we find a strong positive correlation between detrended annual air temperature anomalies and detrended annual biomass carbon anomalies in our BLUE estimates (Supplementary Fig. 7).

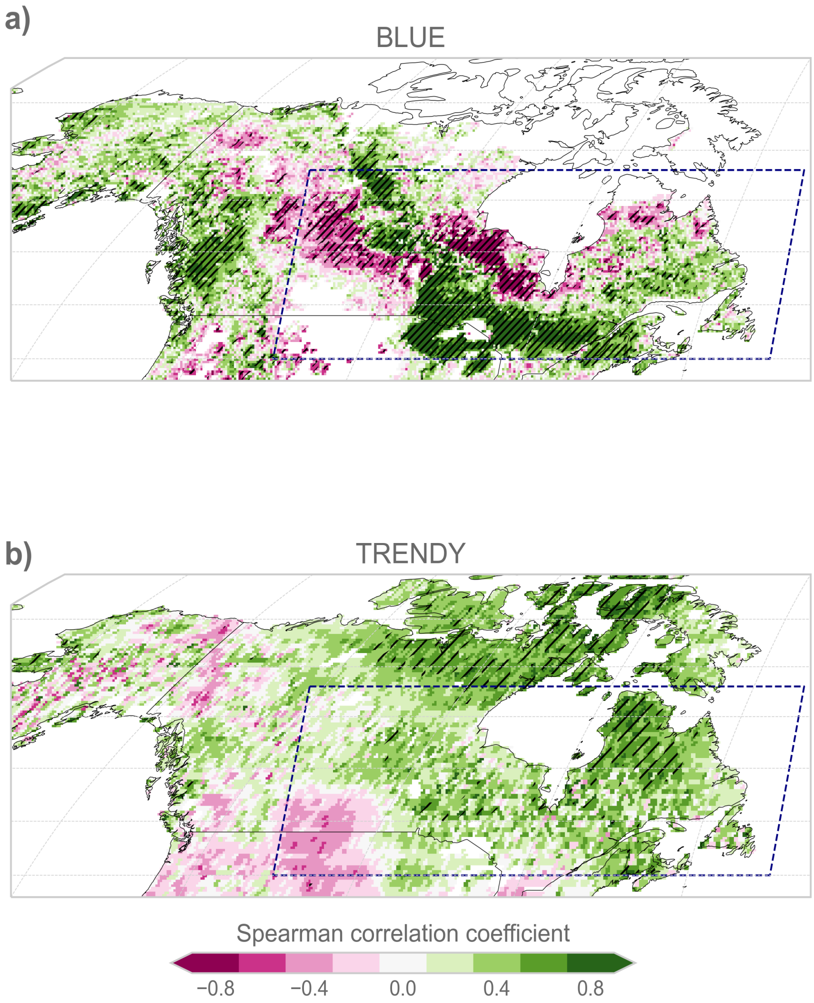

Supplementary Figure 7: Spatial correlation between annual anomalies of climate variables and biomass carbon in North America The maps show the Spearman annual anomalies of climate variables biomass carbon anomalies and the time series of air temperature (Ta) anomalies. The BLUE estimate is shown in a) and the TRENDY estimate (correlation is calculated as average biomass carbon over eight DGVMs from the S3 setup) is shown in b). Significance is tested with a two-tailed t-test and significant correlations ($p<0.05$) are visualized by hatched areas. The dark blue frame denotes parts of the North American boreal forest.

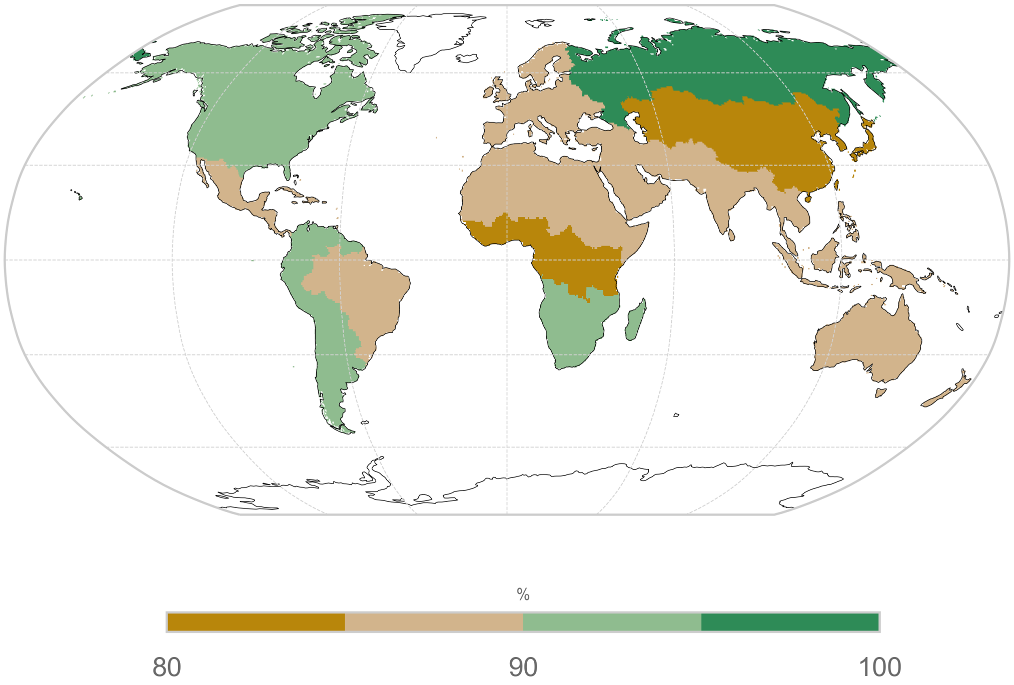

Supplementary Figure 8: Regional accuracy of the data assimilation. The figure shows the regionally averaged spatial agreement between a) the assimilated woody biomass carbon time series and b) the original woody biomass carbon time series by Xu *et al.* [2] for 2000-2019. The agreement (in %) is calculated as the number of years with the same trend (i.e., increase/decrease/no change in biomass carbon) for a) and b) divided by the total number of years.

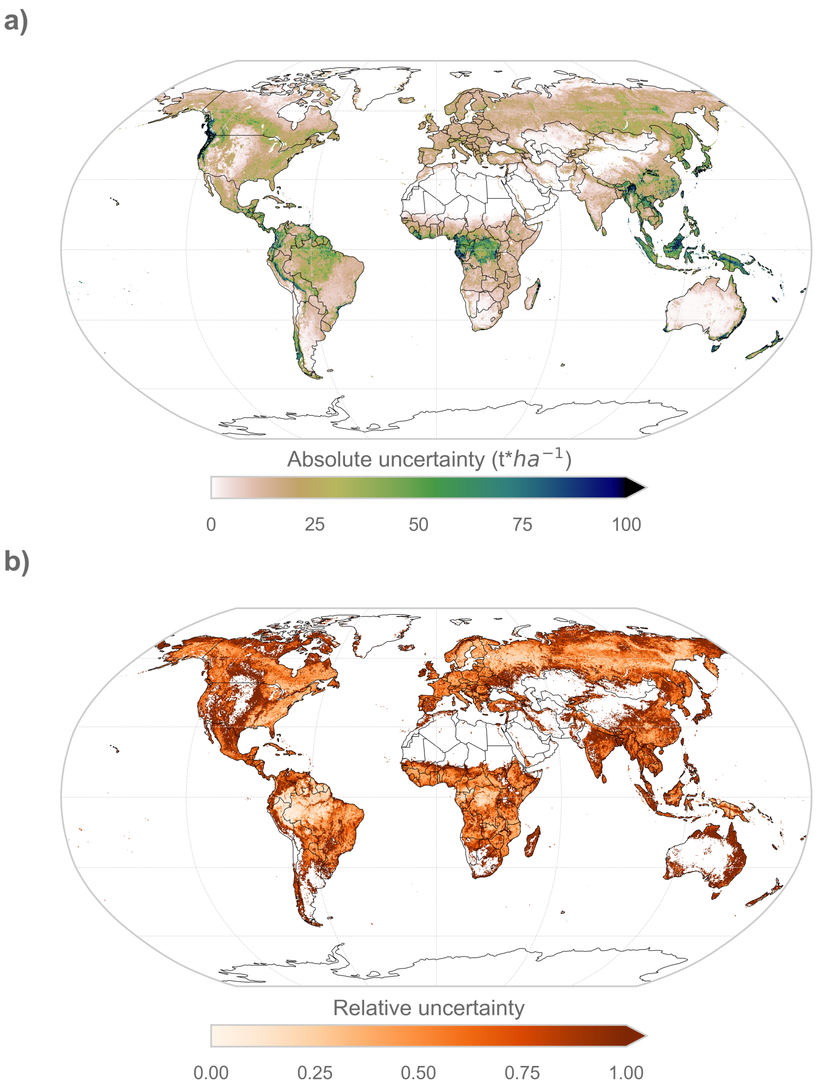

Supplementary Figure 9: Absolute (a) (in tha^{-1}) and relative (b) (as fraction of the original estimate) pixel-level uncertainty for biomass carbon densities, as estimated in Xu *et al.* [2].

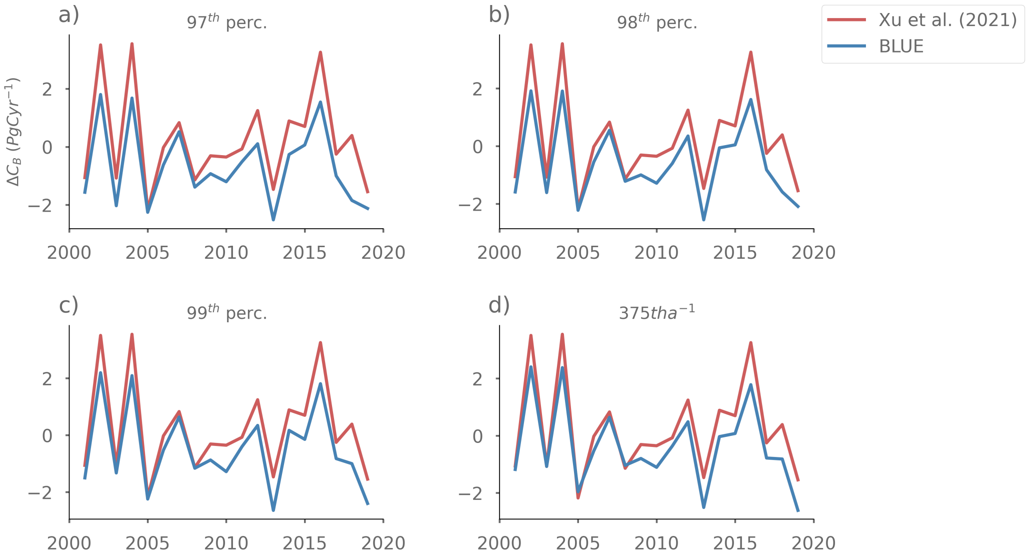

Supplementary Figure 10: Evaluation of different percentile thresholds for excluding unrealistic biomass carbon densities. Comparison of the annual change in global woody biomass carbon stocks (ΔC_B) between the assimilated BLUE time series resulting from different upper thresholds for excluding unrealistic woody biomass carbon densities and the original time series by Xu *et al.* [2]. The percentile-based thresholds are derived by cutting the distribution of woody biomass carbon densities smaller than 375 tha^{-1} to the respective percentiles and choosing the percentile values as upper thresholds for the exclusion of further grid cells. The resulting time series of change in woody biomass carbon stocks from the percentile approaches are shown in a-c and the time series based on an upper threshold of 375 tha^{-1} is shown in d.

**Flux by LULCC transition (PgC yr^{-1})**	**Model simulation**		
	BLUE_transient	BLUE_fixed	BLUE_default
Clearing for pasture and cropland expansion	3.5	2.5	2.7
Net flux from wood harvest	1.4	0.7	0.6
Abandonment of pasture and croplands	-2.1	-1.8	-1.8
Sum	2.8	1.4	1.5

Supplementary Table 1: Comparison of estimated global carbon flux from land use and land cover change for different transitions for 2000-2019. The numbers from this study (BLUE_transient and BLUE_fixed) represent the average from two threshold approaches (see "Threshold testing for excluding inconsistent woody biomass carbon densities").

**Dataset**	**Period**	**Terrestrial biomass carbon sink ($\mathbf{S_{LAND,B}}$)**		
		Cumulative (PgC)	Net flux (PgC yr^{-1})	IAV of net flux
This study *•	2001-2019	-29	-1.5	1.0
Xu *et al.* [2] •	2001-2019	-93	-4.9	0.4
TRENDY v8 (S2) #	2001-2018	-33±9	-1.9±0.5	0.5±0.3
Gasser *et al.* [3]	2000-2018	-22	-1.2	0.8
• Estimate only includes woody biomass carbon				

Supplementary Table 2: Comparison of the estimated global terrestrial biomass carbon sink from this study compared to a range of other recent studies. Interannual variability (IAV) is calculated as the ratio of the standard deviation (SD) to the mean. Error estimates are given as the mean of 13 DGVMs ± 1 SD for TRENDY (#) resp. as the mean from two threshold approaches (see "Threshold testing for excluding inconsistent woody biomass carbon densities") (*). Note that, to avoid errors from the rounding of numbers in the table, percentage values in the main text were calculated from unrounded numbers.

Model	Reference	Available setups
CLASS-CTEM	Melton & Arora [4]	S2,S3,S5,S6
CLM5.0	Lawrence *et al.* [5]	S2
DLEM	Tian *et al.* [6]	S2,S3,S5,S6
JSBACH	Mauritsen *et al.* [7]	S2,S3,S5,S6
JULES-ES 1.02	Sellar *et al.* [8]	S2
LPJ-GUESS	Smith *et al.* [9]	S2,S3,S5,S6
LPX-Bern	Lienert & Joos [10]	S2,S3,S5,S6
OCN	Zaehle *et al.* [11]	S2
ORCHIDEE	Krinner *et al.* [12]	S2,S3,S5,S6
ORCHIDEE CNP	Goll *et al.* [13]	S2,S3,S5,S6
SDGVM	Walker *et al.* [14]	S2,S3,S5,S6
CABLE-POP	Haverd *et al.* [15]	S2
ISAM	Jain *et al.* [16]	S2

Supplementary Table 3: Overview of the simulation setups for the TRENDY DGVMs used in this analysis (adjusted from Obermeier *et al.* [17]).

LULCC transition	Handling Cdens on initially woody vegetation (PFT 1-8)
v to s<br>s to s	Xu et al. (2021) - PFT- and land cover type level<br>threshold approach - linear interpolation
v to c<br>v to p<br>s to c<br>s to p	Xu et al. (2021) - PFT- and land cover type level<br>threshold approach - linear interpolation<br>scaling to c and p acc. to Houghton ratios
c to p<br>p to c<br>c to s<br>p to s	Xu et al. (2021) - PFT- and land cover type level<br>threshold approach - linear interpolation<br>scaling to c and p acc. to Houghton ratios<br>interpolation for grid cells without v/s land cover upon initialization

Supplementary Table 4: Handling of transitions on initially woody vegetation (black fonts show new steps introduced compared to upper rows). The land cover types are abbreviated as v: primary land (in BLUE "virgin"), s: secondary land, c: cropland and p: pasture.

Supplementary Note 1. Assimilation of observed woody biomass carbon in BLUE

Threshold testing for excluding inconsistent woody biomass carbon densities
We compare the assimilated woody biomass carbon densities resulting from different thresholds for excluding unrealistically high values at the beginning of each time step in BLUE to the original woody biomass carbon densities by Xu *et al.* [2]. We test various thresholds to account for uncertainties introduced by the choice of an upper threshold for unrealistic woody biomass carbon densities. The percentile thresholds (97th, 98th and 99th percentile) are applied to each year of the woody biomass carbon density time series that was generated by excluding all values larger than the maximum pixel-value of the time series by Xu *et al.* [2] (i.e., all values equal to or larger than 375 tha^{-1}). All woody biomass carbon densities that are equal to or exceed the chosen percentile threshold are excluded from the dataset and interpolated by means of linear barycentric interpolation. The fraction of excluded grid cells (related to the total number of grid cells: 720x1440 at 0.25°resolution), including grid cells that were already excluded prior to the calculation of percentiles according to the 375 tha^{-1} threshold ($\sim 3\%$), varies from $\sim 4\%$ (99th percentile) to $\sim 6\%$ (97th percentile) over the entire time series. We evaluate each threshold by comparing the global biomass carbon trends (i.e., increase/decrease) of the assimilated time series to the original time series. Supplementary Fig. 10 shows that the trends of the assimilated woody biomass carbon time series start to diverge from the observed woody biomass carbon time series in recent years (especially 2014-2015 and 2017-2018) for thresholds smaller than the 99th percentile. Consequently, we choose the 99th percentile as an additional threshold approach to the 375 tha^{-1} approach.

Special assumptions

Upon any LULCC transition, the woody biomass carbon density of the target cover type is zero if the area covered by the target cover type was zero upon initialization of BLUE. Consequently, - albeit a change in land use and/or land cover can occur at a later time - there would be no carbon transferred from the source land cover type to the target land cover type in the respective PFT. In those special cases, the actual biomass carbon density for the target land cover type in the respective PFT is calculated by scaling the actual biomass carbon density from the source land cover type in the respective PFT with the relation of source and target land cover type, taken from the Houghton *et al.* [18] carbon densities:

$$\rho_{Ba,j',l}(t) = \rho_{Ba,j,l}(t) * \frac{\rho_{B,j',l}}{\rho_{B,j,l}} \quad (1)$$

with $j\{v, s, p, c\}; l\{1..8\}; t\{2000..2019\}$

Handling of non-woody vegetation

Non-woody vegetation carbon (i.e., PFTs 9..11 or land cover type p,c) is excluded (i.e., initialized with zero) for non-woody PFTs in our simulation setups. The inclusion of carbon fluxes on non-woody vegetation types might be desirable to obtain holistic values for some carbon fluxes or to facilitate the comparison with other datasets (mainly relevant for E_{LUC} and C_B in our analysis). For those variables, we add the non-woody carbon fluxes from the "default" BLUE simulations with woody biomass carbon densities based on Houghton *et al.* [18] (but otherwise the same setup).

However, there is still non-woody vegetation which needs to be directly considered in our assimilated biomass carbon setups. This is the case for areas that are naturally vegetated by woody biomass (PFTs 1..8), but that were converted to non-woody land cover types (p,c) through LULCC and can return to woody vegetation again any time through abandonment (i.e., pasture/crop is converted to secondary land). Similarly, woody vegetation might be temporarily converted to non-woody vegetation through clearing for agriculture or pasture. Consequently, we need biomass carbon density estimates for non-woody vegetation on areas where woody vegetation naturally grows. This is achieved by 1) scaling the "processed" (i.e., threshold approach and interpolation finished) woody biomass carbon densities for primary and secondary land to crop and pasture according to the ratios from Houghton *et al.* [18] and 2) interpolating "missing" values, i.e., grid cells with no primary or secondary land cover upon initialization (only 1% of all grid cells over the entire time series). Supplementary Tab. 4 gives an overview of how each LULCC transition type is realized.

References

1. Pan, Y. *et al.* A large and persistent carbon sink in the world's forests. *Science* **333,** 988–93. ISSN: 1095-9203 (Electronic) 0036-8075 (Linking) (2011).
2. Xu, L. *et al.* Changes in global terrestrial live biomass over the 21st century. *Sci Adv* **7.** ISSN: 2375-2548 (Electronic) 2375-2548 (Linking) (2021).
3. Gasser, T. *et al.* Historical CO_2 emissions from land use and land cover change and their uncertainty. *Biogeosciences* **17,** 4075–4101. ISSN: 1726-4189 (2020).
4. Melton, J. R. & Arora, V. K. Competition between plant functional types in the Canadian Terrestrial Ecosystem Model (CTEM) v.2.0. *Geoscientific Model Development* **9,** 323–361. ISSN: 1991-9603 (2016).
5. Lawrence, D. M. *et al.* The Community Land Model Version 5: Description of New Features, Benchmarking, and Impact of Forcing Uncertainty. *Journal of Advances in Modeling Earth Systems* **11,** 4245–4287. ISSN: 1942-2466 1942-2466 (2019).
6. Tian, H. *et al.* North American terrestrial CO2 uptake largely offset by CH4 and N2O emissions: toward a full accounting of the greenhouse gas budget. *Clim Change* **129,** 413–426. ISSN: 0165-0009 (Print) 0165-0009 (Linking) (2015).
7. Mauritsen, T. *et al.* Developments in the MPI-M Earth System Model version 1.2 (MPI-ESM1.2) and Its Response to Increasing CO2. *J Adv Model Earth Syst* **11,** 998–1038. ISSN: 1942-2466 (Print) 1942-2466 (Linking) (2019).
8. Sellar, A. A. *et al.* UKESM1: Description and Evaluation of the U.K. Earth System Model. *Journal of Advances in Modeling Earth Systems* **11,** 4513–4558. ISSN: 1942-2466 1942-2466 (2019).
9. Smith, B. *et al.* Implications of incorporating N cycling and N limitations on primary production in an individual-based dynamic vegetation model. *Biogeosciences* **11,** 2027–2054. ISSN: 1726-4189 (2014).
10. Lienert, S. & Joos, F. A Bayesian ensemble data assimilation to constrain model parameters and land-use carbon emissions. *Biogeosciences* **15,** 2909–2930. ISSN: 1726-4189 (2018).

11. Zaehle, S., Ciais, P., Friend, A. D. & Prieur, V. Carbon benefits of anthropogenic reactive nitrogen offset by nitrous oxide emissions. *Nature Geoscience* **4,** 601–605. ISSN: 1752-0894 1752-0908 (2011).

12. Krinner, G. *et al.* A dynamic global vegetation model for studies of the coupled atmosphere-biosphere system. *Global Biogeochemical Cycles* **19.** ISSN: 08866236 (2005).

13. Goll, D. S. *et al.* A representation of the phosphorus cycle for ORCHIDEE (revision 4520). *Geoscientific Model Development* **10,** 3745–3770. ISSN: 1991-9603 (2017).

14. Walker, A. P. *et al.* The impact of alternative trait-scaling hypotheses for the maximum photosynthetic carboxylation rate (Vcmax) on global gross primary production. *New Phytol* **215,** 1370–1386. ISSN: 1469-8137 (Electronic) 0028-646X (Linking) (2017).

15. Haverd, V., Smith, B., Nieradzik, L. P. & Briggs, P. R. A stand-alone tree demography and landscape structure module for Earth system models: integration with inventory data from temperate and boreal forests. *Biogeosciences* **11,** 4039–4055. ISSN: 1726-4189 (2014).

16. Jain, A. K., Kheshgi, H. S. & Wuebbles, D. J. A globally aggregated reconstruction of cycles of carbon and its isotopes. *Tellus B: Chemical and Physical Meteorology* **48,** 583–600. ISSN: 1600-0889 (2017).

17. Obermeier, W. A. *et al.* Modelled land use and land cover change emissions – a spatio-temporal comparison of different approaches. *Earth System Dynamics* **12,** 635–670. ISSN: 2190-4987 (2021).

18. Houghton, R. A. *et al.* Changes in the Carbon Content of Terrestrial Biota and Soils between 1860 and 1980: A Net Release of CO"2 to the Atmosphere. *Ecological Monographs* **53,** 235–262. ISSN: 0012-9615 1557-7015 (1983).
